# Inward Outward Signaling in Ovarian Cancer: Morpho-Phospho-Proteomic Profiling Upon Application of Hypoxia and Shear Stress Characterizes the Adaptive Plasticity of OVCAR-3 and SKOV-3 Cells

**DOI:** 10.3389/fonc.2021.746411

**Published:** 2022-02-14

**Authors:** Andrea Bileck, Patricia Bortel, Michelle Kriz, Lukas Janker, Endre Kiss, Christopher Gerner, Giorgia Del Favero

**Affiliations:** ^1^ Department of Analytical Chemistry, Faculty of Chemistry University of Vienna, Vienna, Austria; ^2^ Joint Metabolome Facility, University of Vienna and Medical University of Vienna, Vienna, Austria; ^3^ Department of Food Chemistry and Toxicology, Faculty of Chemistry University of Vienna, Vienna, Austria; ^4^ Core Facility Multimodal Imaging, Faculty of Chemistry University of Vienna, Vienna, Austria

**Keywords:** ovarian cancer, fluid shear stress (FSS), hypoxia, morpho-metabolic plasticity, vasculogenic mimicry (VM)

## Abstract

With the onset of resistance, ovarian cancer cells display almost unpredictable adaptive potential. This may derive from the tumor genetic ancestry and can be additionally tailored by post translational protein modifications (PTMs). In this study, we took advantage of high-end (phospho)-proteome analysis combined with multiparametric morphometric profiling in high-grade serous (OVCAR-3) and non-serous (SKOV-3) ovarian carcinoma cells. For functional experiments, we applied two different protocols, representing typical conditions of the abdominal cavity and of the growing tumor tissue: on the one side hypoxia (oxygen 1%) which develops within the tumor mass or is experienced during migration/extravasation in non-vascularized areas. On the other hand, fluid shear stress (250 rpm, 2.8 dyn/cm^2^) which affects tumor surface in the peritoneum or metastases in the bloodstream. After 3 hours incubation, treatment groups were clearly distinguishable by PCA analysis. Whereas basal proteome profiles of OVCAR-3 and SKOV-3 cells appeared almost unchanged, phosphoproteome analysis revealed multiple regulatory events. These affected primarily cellular structure and proliferative potential and consolidated in the proteome signature after 24h treatment. Upon oxygen reduction, metabolism switched toward glycolysis (e.g. upregulation hexokinase-2; HK2) and cell size increased, in concerted regulation of pathways related to Rho-GTPases and/or cytoskeletal elements, resembling a vasculogenic mimicry response. Shear stress regulated proteins governing cell cycle and structure, as well as the lipid metabolism machinery including the delta(14)-sterol reductase, kinesin-like proteins (KIF-22/20A) and the actin-related protein 2/3 complex. Independent microscopy-based validation experiments confirmed cell-type specific morphometric responses. In conclusion, we established a robust workflow enabling the description of the adaptive potential of ovarian cancer cells to physical and chemical stressors typical for the abdominal cavity and supporting the identification of novel molecular mechanisms sustaining tumor plasticity and pharmacologic resistance.

## Introduction

Ovarian cancers develop in rather aggressive phenotypes which reflects in the highest fatality rate among gynecological tumors (1). This is surely attributable to remarkable heterogeneity of the tumor variants combined with high inter-individual variability (2). In addition to genetic ancestry, post-translational protein modifications and metabolic properties are increasingly recognized as crucial contributors in shaping the heterogeneity of the disease. As an example, it was recently demonstrated that lipids and calcium management sustain pathologic progression of ovarian cancer and fuel resistance *in vivo* and *in vitro* (3). In addition, ovarian cancer cells are capable of metabolic and functional switching to different states (also described as miliary and non-miliary metastatic forms), and these fluctuations are associated with different responsiveness to therapy (4, 5).

In addition to biochemical pathways, the role of biomechanical stimulation and mechanotransduction is emerging in the study of tumor pathophysiology (6–8). It is now clear that physical cues can contribute to the regulation of multiple pathways, including the cellular metabolic state, such as glycolysis (9, 10) and lipid metabolism (11). These aspects are particularly relevant for ovarian cancer (12) hence the development in the peritoneal cavity necessarily requires endurance to physical stressors. These originates, among others, from the movement of the intestine, the fluid shear stress of the luminal fluids or the development of ascites (13). Most crucially, physical stimuli may support the progression of the disease: even without colonizing other tissues, ovarian cancer can spread broadly in the abdominal cavity (13, 14): this is eased by the formation of spheroids that are growing and disseminating with the ascitic fluids (15). In this respect, recent studies correlated ovarian cancer cell morphology/survival to the capability to cope with biomechanical stimulation (16–18). Moreover, shear stress was found to modulate cytotoxicity/resistance of cisplatin and carboplatin in tumor spheroids (19, 20). However, literature is still limited and the elucidation of the molecular mechanisms influencing these responses is far from being complete.

From a cellular perspective, proliferation in peritoneum relates to mechanotransduction, including, maintenance of energetic balance, anchoring capacity and the formation of 3D structures even under flow. In addition, metabolic plasticity is necessary to cope with reduced oxygen (hypoxia) and limited access to vascular supply. Indeed, hypoxia plays a recognized role in driving resistance of ovarian cancer cells (21) and its regulatory potential expands extensively from the modification of cell morphology to ECM remodeling (22, 23). Indeed, as complementary faces of the same medal, both hypoxia and mechanotransduction are capable of governing structural and metabolic adaptation pathways. Building on this, we took advantage of multiparametric structural characterization and comprehensive untargeted proteome and phosphoproteome profiling to compare the adaptive potential of two commonly used ovarian cancer cell models, namely OVCAR-3 and SKOV-3. Even with the obvious exemplification of *in vitro* models, the two cell types are representative of different pathological states, namely high-grade serous carcinomas, spreading broadly in the peritoneal cavity (OVCAR-3) and non-serous carcinomas (SKOV-3), which are characterized by high migratory behavior (24). Thanks to this approach, we could systematically apply different workflows, reproducing hypoxia and shear stress as typical stimuli benchmarking ovarian cancer growth and progression in the peritoneum. Untargeted analysis allowed us to identify the similarities and the differences of the individual signatures of SKOV-3 and OVCAR-3 and to dissect the potential contribution of physical and chemical extracellular stimuli in pathways decisive for resistance and tumor cell plasticity.

## Materials and Methods

### Cell Culture

For the purpose of this study two commercially available human ovarian epithelial adenocarcinoma cell lines were used (SKOV-3 and OVCAR-3). Both cell types were acquired from the American Type Culture Collection (ATCC, Manassas, Virginia, USA) and cultivated according to the specification of the supplier in base media supplemented with heat-inactivated fetal calf/bovine serum (FCS, Gibco, Thermo Fisher Scientific, Austria) and maintained in humidified incubators (37°C and 5% CO_2_). SKOV-3 cells were cultivated in McCoy’s 5a Medium Modified (Gibco, Thermo Fisher Scientific, Austria) with 10% (v/v) FCS and 1% (v/v) penicillin-streptomycin (P/S, Sigma-Aldrich, Austria), and OVCAR-3 cells were cultivated in RPMI-1640 Medium (Gibco, Thermo Fisher Scientific, Austria) supplemented with 20% (v/v) FCS, 1% (v/v) P/S and 1% (w/v) insulin-transferrin-selenium (ITS-G, 100X, Gibco, Thermo Fisher Scientific). Cell number was determined with a MOXI Z Mini Automated Cell Counter with the corresponding MOXI Z Type M Cassettes (ORFLO Technologies, USA) and experiments were performed with cells reaching a confluency of 80%.

### Incubations and Experimental Layout

For the experiments, cells were seeded into 6-well plates for adherent cells (Sarstedt, Austria) with 150,000 cells/well (SKOV-3) and 300,000 cells/well (OVCAR-3). At the beginning and at the end of the experiments cells were imaged (bright field and phase contrast modes) with a Lionheart FX Automated Microscope (BioTek Instruments Winooski, VT, USA). Control cells were placed in the standard incubator (normal oxygen: NO, static: ST; 20% O_2_). Reduced oxygen (RO; hypoxia) was obtained with the Lionheart microscope equipped with a CO_2_/O_2_ gas controller by flushing the incubation chamber with N2 (1% O_2_ and 94% N_2_). Shear stress stimulation was obtained with the orbital shaker method (25). Plates were maintained in 20% O_2_-5% CO_2_ atmosphere and incubated with an orbital shaker at 250 rpm. This value was chosen on the basis of stress response experiments performed in our labs and corresponds to estimated fluid shear stress of approximately 2.8 dyn/cm^2^ (25) which is in line with previous work that described the response of ovarian cancer cells to shear stress [3 dyn/cm^2^ (20)]. For the proteome profiles, 4-6 biological replicates were analyzed for each treatment group. For the phosphoproteome analysis, experiments were performed in triplicate.

### Live Cell Imaging and Image Analysis

All 6-well plates were imaged with the Lionheart microscope acquiring at least 4 optical fields from the central region of the wells and using Gen5 Software Feature for Imaging & Microscopy (10X magnification). Image analysis was performed with the free software ImageJ as previously described (26, 27). For the morphometric profiling, at least 5 representative cells were evaluated from randomly chosen optical fields and at least 50 cells were quantified for every experimental condition in terms of area, perimeter, major and minor axis, circularity and roundness. Mitochondrial staining was obtained as previously described (28) by diluting MitoTracker™ Green (1:1000) in the respective base media for 20 min in the dark. Cell nuclei were counterstained with Hoechst 33258. Afterwards the staining solution and the medium from the unstained wells were removed, wells were rinsed with pre-warmed PBS and cells were maintained in fresh complete medium. Image analysis was performed from at least 3 cell preparations quantifying the integrated density *per* cell from n > 50 cells with free software ImageJ. Data comparison was performed with a two sample t-Test and cutoff (p value) inferior to 0.05.

### Proteomics and Phosphoproteomics

Phosphoproteomic samples were prepared using a previously described protocol (29), employing a slightly modified version of the EasyPhos platform (30). In short, cells were scraped with 4% SDC buffer, heat-treated at 95°C and lysed *via* ultrasonication. Protein concentrations were determined *via* BCA-assay. 200 µg of protein was reduced and alkylated with TCEP and 2-CAM, followed by enzymatic digestion overnight with Trypsin/Lys-C (1:100 Enzyme to Substrate ratio, room temperature). Digested samples were mixed with enrichment buffer and incubated with TiO_2_ Titansphere beads (GL Sciences) for phosphopeptide enrichment. After sample clean-up *via* C_18_ StageTips, phosphopeptides were eluted, dried and reconstituted in MS loading buffer.

Preparation of cytoplasmic and nuclear fractions of proteomic samples was performed according to a previously described workflow (31). In short, cells were lysed in hypotonic buffer supplemented with protease inhibitors applying mechanical shear stress. Cytoplasmic fractions were separated *via* centrifugation and precipitated overnight with ethanol. Nuclei were swelled in extraction buffer, diluted 1:10 with NP-40 buffer and lysates were centrifuged to obtain the nuclear proteins which were further precipitated overnight with ethanol. After precipitation, all samples were dissolved in lysis buffer (8M Urea, 1M TEAB, 20% (w/v) SDS) and BCA-assay was performed to determine protein concentrations.

A Protifi S-trap digestion protocol (32) was applied for the preparation of the proteomic samples, including the whole cell lysate global proteome samples. Whole cell lysate global proteome samples were solubilized with 5% SDS prior to S-trap processing. 20 µg of protein was reduced with DTT, alkylated with IAA, acidified and mixed with trapping buffer. Samples were loaded onto S-trap columns, washed and digested with Trypsin/Lys-C for 4 h (1:40 Enzyme to Substrate ratio, 37°C). Peptides were eluted, dried, reconstituted in 30% formic acid containing synthetic standard peptides and diluted with mobile phase A.

LC-MS/MS analyses were performed using a timsTOF pro mass spectrometer (Bruker Daltonics) hyphenated with a Dionex Ultimate 3000 nano LC-system (Thermo Fisher Scientific). Measurement conditions for LC and MS were an adapted version of a recently published method (33).

**Figure 1:**
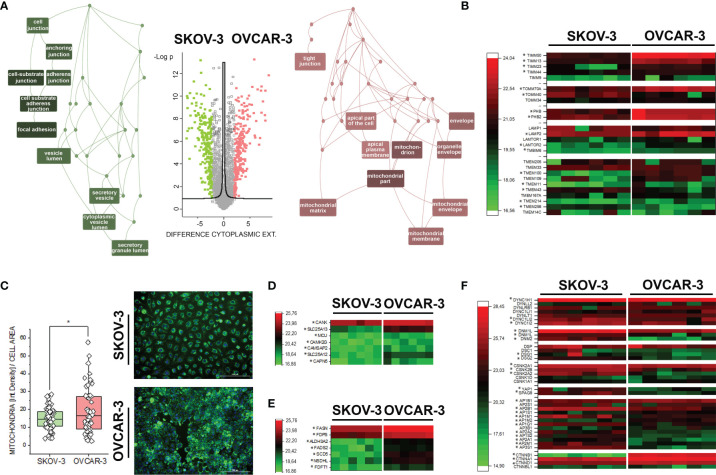
Proteome profile of SKOV-3 and OVCAR-3. **(A)** Volcano plot displaying differentially regulated proteins in the cytoplasmic compartment (FDR < 0.05). Enriched GO terms graphs (WebGestalt (42) of the cellular components underpinning the regulatory events: depicted in pink upregulated proteins in OVCAR-3 cells; green upregulated proteins in SKOV-3 cells. **(B)** Heat map summarizing differentially regulated mitochondrial-autophagy proteins in OVCAR-3 and SKOV-3. **(C)** Mitochondrial morphology (green, MitoTracker) and signal intensity per cell (n > 50 cells, * significant difference t-Test p < 0.05). Heat maps summarizing differentially regulated proteins related to calcium management **(D)**, lipid biosynthesis **(E)**, selected cytoskeletal elements and transcription factors **(F)** in OVCAR-3 and SKOV-3. Columns in the heat maps are depicting biological replicates (n = 6) and *significant regulations FDR < 0.05.

**Figure 2:**
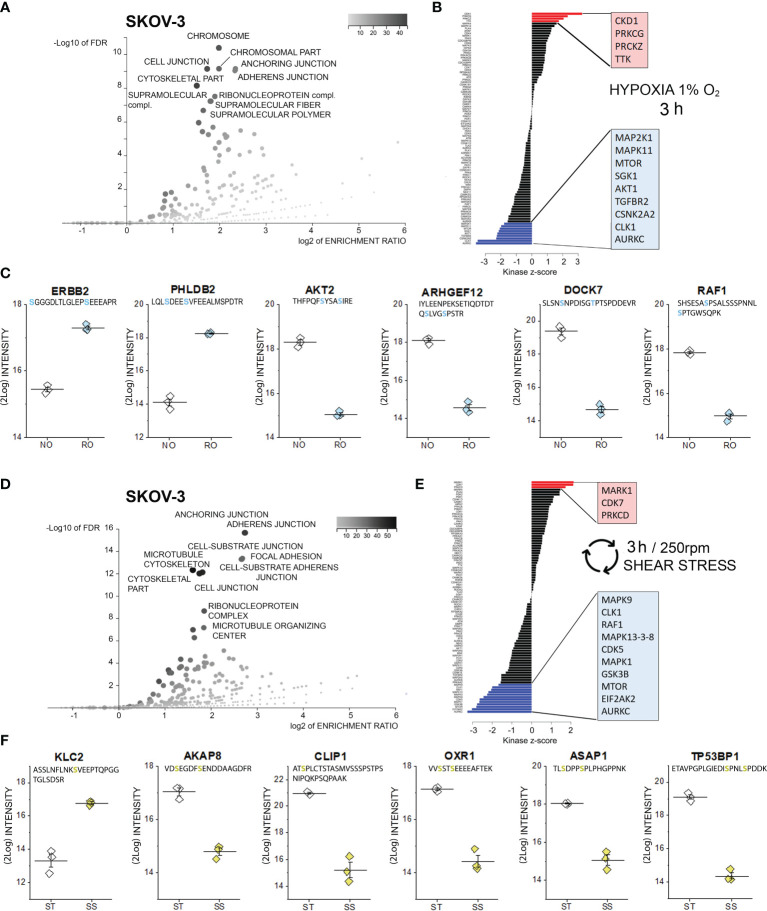
Phosphoproteome profile of SKOV-3 cells. **(A)** Cellular components primarily targeted by phosphorylation events after 3h hypoxia incubation [volcano plot generated with WebGestalt (42) and adapted]. **(B)** Waterfall plot depicting the association between kinases and phosphorylation events modified by hypoxia incubation. Substrate enrichment in comparison to controls is indicated in red, substrate decline is indicated in blue. **(C)** Representative proteins with modified phosphorylation patterns, peptide sequences are indicated below the protein names and residues depicted in light blue identify phospho (STY) probabilities > 0.75; NO Normal Oxygen (20%, white diamonds); RO Reduced Oxygen (1%, hypoxia, light blue diamonds) difference between treatments groups p-value < 0.0001 (Student’s t-Test). **(D)** Cellular components primarily targeted by phosphorylation after 3h shear stress [volcano plot generated with WebGestalt (42) and adapted]. **(E)** Waterfall plot depicting the association between kinases and phosphorylation events modified by shear stress incubation. Substrate enrichment in comparison to controls is indicated in red, substrate decline is indicated in blue. **(F)** Representative proteins with modified phosphorylation patterns, peptide sequences are indicated below the protein names and residues depicted in yellow identify phospho (STY) probabilities > 0.75; ST static incubation (white diamonds), SS shear stress (250 rpm, 2.8 dyn/cm^2^, yellow diamonds) difference between treatments groups p-value < 0.01 (Student’s t-Test).

**Figure 3:**
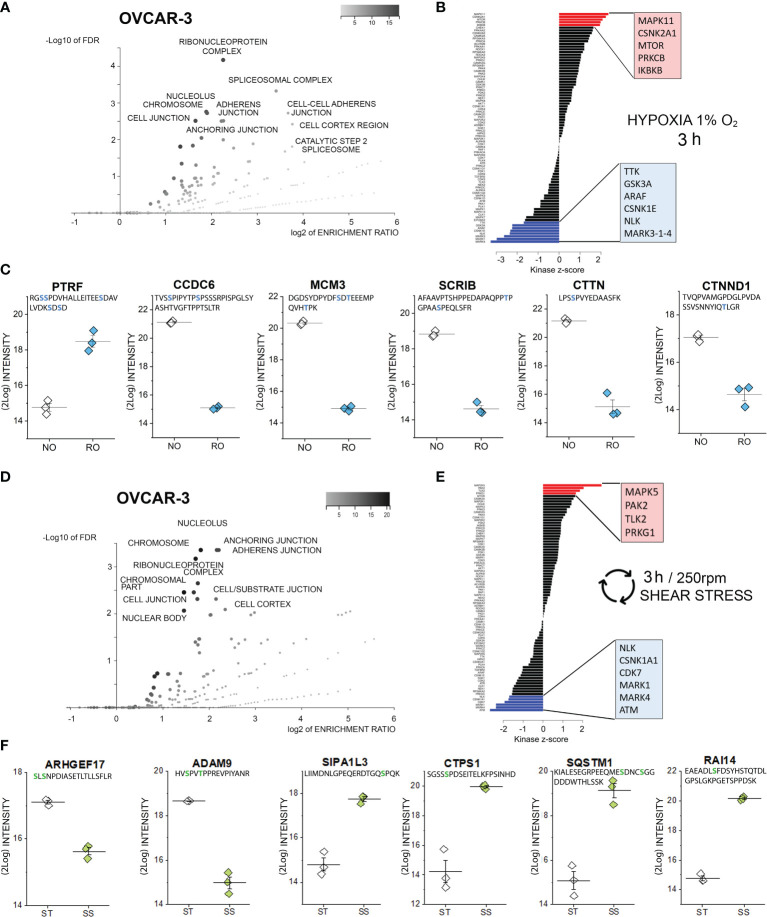
Phosphoproteome profile of OVCAR-3 cells. **(A)** Cellular components primarily targeted by phosphorylation events after 3h hypoxia incubation [volcano plot generated with WebGestalt (42) and adapted]. **(B)** Waterfall plot depicting the association between kinases and phosphorylation events modified by hypoxia incubation. Substrate enrichment in comparison to controls is indicated in red, substrate decline is indicated in blue. **(C)** Representative proteins with modified phosphorylation patterns; peptide sequences are indicated below the protein names and residues depicted in blue identify phospho (STY) probabilities > 0.75. NO Normal Oxygen (20%, white diamonds); RO Reduced Oxygen (1%, hypoxia, blue diamonds) difference between treatments groups p-value < 0.001 (Student’s t-Test). **(D)** Cellular components primarily targeted by phosphorylation events after 3h shear stress [volcano plot generated with WebGestalt (42) and adapted]. **(E)** Waterfall plot depicting the association between kinases and phosphorylation events modified by shear stress incubation. Substrate enrichment in comparison to controls is indicated in red, substrate decline is indicated in blue. **(F)** Representative proteins with modified phosphorylation patterns; peptide sequences are indicated below the protein names and residues depicted in green identify phospho (STY) probabilities > 0.75. ST static incubation (white diamonds), SS shear stress (250 rpm, 2.8 dyn/cm^2^, green diamonds) difference between treatments groups p-value < 0.001 (Student’s t-Test).

Protein identification and data analysis were performed by means of MaxQuant (version 1.6.17.0) (34) and Perseus (version 1.6.17.0) (35, 36) using the UniProt Database (version 12/2019 with 20 380 entries), allowing a mass tolerance of 20ppm for MS spectra and 40ppm for MS/MS spectra, a FDR < 0.01 and a maximum of 2 missed cleavages. Furthermore, search criteria included carbamidomethylation of cysteine as fixed modification and methionine oxidation, N-terminal protein acetylation as well as phosphorylation of serine, threonine and tyrosine as variable modifications. For the generation of the protein-protein association networks (**Figures 6**, **7**) the STRING database was used (37) and results were integrated in the figures according to the Creative Commons BY 4.0’ license. Proteins outside the primary clusters were eliminated to increase readability. For the interpretation of phosphoproteomics data, a kinase-substrate enrichment analysis of class 1 phosphosites (p > 0.75) utilizing PhosphoSitePlus and NetworKIN was performed, applying a NetworKIN score cutoff of 2, p-value cutoff of 0.05 and substrate count cutoff of 3 (38–40). For the visualization of enriched kinases in context of the global kinome, the application Coral was used (41) (**Supplementary Figure S5**). For the identification of the cellular components primarily affected by regulatory events (**Figures 1A**, **2A/D** and **3A/D** and **Supplementary Figure S1**) enrichment analysis (Over representation analysis, ORA) was performed with WebGestalt platform version 2019 (42–45) interrogating the 250 proteins mostly regulated (**Figure 1A**) or proteins related to phosphorylation events (p < 0.05, **Figures 2** and **3**). Graphs were formally adapted to increase readability. Labels in the volcano plots for the ORA indicate categories with FDR <= 0.05.

### Confocal Microscopy

For the confocal microscopy cells were seeded in petri dishes with 180 µm (+10/-5 µm) thick bottom. This approach allowed us to perform validation experiments cultivating the cells in comparable vessels as for the proteome-phosphoproteome analysis, including medium/cell density ratio and geometrical parameters which are essential for reproducible shear stress application. After 3 hours incubation cells were fixed in pre-warmed formaldehyde (3.7%, 37°C). Afterwards, cells were permeabilized with triton X-100 (0,2%, 15 min) and unspecific reactive sites were blocked with 2% normal goat serum (Sigma-Aldrich) for 1 hour at room temperature. Actin cytoskeleton was marked with Oregon Green 488 phalloidin (Molecular Probes, depicted in green, 1:500 dilution, 1hour incubation at room temperature) and cell nuclei counterstained with DAPI (depicted in blue). Petri dishes were mounted with Mounting Medium with DAPI - Aqueous, Fluoroshield (Abcam). Single plane XY-scanned images were acquired with a Zeiss LSM710 confocal microscope with a Plan-Apochromat 63X/1.4 oil immersion DIC objective. For every experimental condition, samples were prepared in triplicates and images were acquired from minimum 3 different optical fields. Analysis was performed on n > 45 cells after background correction and maintaining acquisition parameters signal/noise ratio constant. Fluorophore signals were measured as mean relative fluorescence units (r.f.u.) and quantified with the software ZEN Zeiss (black edition). Data comparison was performed with a two-sample t-Test and cutoff (p value) inferior to 0.05.

## Results

### Proteome Profile of OVCAR-3 and SKOV-3: Morpho-Metabolic Differences

Characterization of proteome signatures of SKOV-3 and OVCAR-3 confirmed that the clones of our laboratories displayed the typical features representing their pathological model. Six independent replicates were analyzed per treatment condition and cell fraction with Pearson correlation coefficients typically above 90%, ranging from 0.85 to 0.96 between replicates. In total, 3324 proteins were identified with at least two peptides per protein, in at least 5 out of six replicates in one group and applying a false discovery rate (FDR) of less than 1% at both protein and peptide level. Comparative proteome analysis of the nuclear extracts revealed 348 differently expressed proteins, i.e. more than two-fold and significantly regulated between the two cell types (FDR < 0.05) (**Supplementary Figure S1** and **Supplementary Data**). Concerning the cytoplasmic fraction, 1160 proteins differed more than two-fold and significantly between the two cell types (FDR < 0.05). OVCAR-3 showed significantly higher expression of epithelial marker proteins such as KRT18, Claudins 3 and 6, EPCAM, whereas SKOV-3 displayed higher levels of stem cell marker proteins CD44, L1CAM and KRT19 as well as EMT marker vimentin, ERBB2 and IGFBP7 (**Supplementary Figure S2**). ORA enrichment analysis (42) for OVCAR-3 indicated mitochondria and the apical part of the cell/plasma membrane as the cellular components primarily abundant in comparison to SKOV-3 (**Figure 1A**). This was exemplified by the regulation of multiple mitochondria import proteins (TIMM and TOMM, **Figure 1B**) (46, 47). Differences in the subcellular distribution of mitochondria could be confirmed independently *via* live cell imaging, where the organelles appeared more branched in SKOV-3 cells and rather clustered in the perinuclear compartment in OVCAR-3 (**Figure 1C**). In addition, quantification of the mitochondrial fluorescence signal/cell showed a significant prevalence for the OVCAR-3 in comparison to the SKOV-3 (**Figure 1C**). Coherently, prohibitins, which regulates mitophagy (48), were found upregulated in OVCAR-3, together with the Lysosome-associated membrane glycoprotein 2 (LAMP2) which is essential for autophagy mediated mitochondrial turnover (49, 50) (**Figure 1B**). In line with the differences postulated by the mitochondrial signature, the upregulation of several calcium binding proteins such as the calcium-binding mitochondrial carrier protein Aralar1 and 2 (SLC25A12; SLC25A13), and calcium uniporter protein, mitochondrial (MCU) was detected (**Figure 1D**). In addition, structural elements significantly endowed the differences between OVCAR-3 and SKOV-3. Adhesion, junctional proteins and cytoskeletal elements appeared more expressed in the SKOV-3 cells (**Figure 1A**), including, among others, several dynamins, dyneins and tubulin isoforms, desmoglein 1 and 2, L-1 cell adhesion molecule L1CAM, as well as the casein kinase II subunits alpha and beta (**Figure 1F** and **Supplementary Material**). This picture is in line with the high motility typically displayed by SKOV-3 *in vitro* (24) and further supported by the high expression of YAP1, a mechanosensitive transcription factor which mediates cell response to physical cues [(51–53) **Figure 1F**]. Particular for the signature of OVCAR-3 was the consistent high expression of enzymes involved in lipid metabolism, particularly cholesterol biosynthesis [e.g. squalene synthase, FDFT1; farnesyl pyrophosphate synthase, FDPS (54) **Figure 1E**]. In agreement with the boost in lipid metabolism, a marked regulation of catenin beta [CTNNB1 (55)] was also observed (**Figure 1F**).

### Proteome Profile of SKOV-3 and OVCAR-3 Cells After 3h Incubation in Reduced Oxygen or Shear Stress

All protein regulatory events reported in the following paragraphs are significant applying a false discovery rate of a maximum of 5%. Rapid adaptive responses to hypoxia and shear stress (three hours incubation) were evaluated *via* proteome and phosphoproteome analysis. Proteome analysis showed minimal changes following hypoxia or shear stress stimulation: one protein was downregulated in SKOV-3 cells for every experimental condition. Incubation in reduced oxygen decreased the detection of the RAC-beta serine/threonine-protein kinase (AKT2), which was probably related to the consistent de-phosphorylation of the protein (**Supplementary Figure S3**). Shear stress treatment significantly downregulated the Zinc finger CCCH domain-containing protein 4 (ZC3H4). For OVCAR-3 two proteins were found downregulated after hypoxia incubation, namely the cytoskeletal component coiled-coil domain-containing protein 6 (CCDC6) and the DNA replication licensing factor MCM3, which is essential for cell proliferation. Intriguingly, OVCAR-3 responded to the 3h shear stress incubation protocol by upregulating the protein ankycorbin (RAI14), which mediates actin regulation (**Supplementary Figure S4**).

### Phosphoproteome Profile of SKOV-3 Cells After 3h Incubation in Reduced Oxygen or Shear Stress

In order to elucidate the leading molecular events initiating cellular adaptation to hypoxia and shear stress, phosphoproteome analyses were performed. Phosphopeptides were enriched with Titansphere beads from whole cell lysates and analyzed using an ion mobility high-resolution time of flight mass spectrometer as described in the Methods section. The following analysis of identified phosphopeptides was restricted to those meeting an identification false discovery rate of 1% and a phosphorylation site confidence of at least 75%. Thus, a total of 2978 phosphopeptides were identified in case of OVCAR-3 cells and total of 3094 phosphopeptides in case of SKOV-3 cells. Two-sided Student t tests were applied to assess phosphorylation and dephosphorylation events with p-values better than 0.05. Treatment-associated abundance differences in phosphopeptides were mapped to known kinase target specificities and signaling pathways as described in the Methods section. Using this approach, indeed multiple regulatory events were detectable after 3h incubation in SKOV-3 cells. According to the enrichment analysis (Gene Ontology category “cellular components”), proteins associated to the terms “chromosome” and several structural elements were mostly affected (3h hypoxia stimulation, **Figure 2A**). Along this line, kinase-substrate enrichment analysis of the phosphorylated proteins traced them back to a significant activation of the cyclin-dependent kinase 1, which is essential in the regulation of cell cycle and microtubules reorganization in mitosis (56). A link toward the regulation of cell proliferation was reinforced by the activation of two isoforms of the protein kinase C (PRKC) gamma and zeta type and dual specificity protein kinase TTK. On a similar note, de-phosphorylation events involved multiple kinase pathways also potentially governing cell proliferation; these included the mitogen-activated protein kinases (MAPK11, MAP2K1), mTOR, serine/threonine-protein kinase Sgk1, RAC-alpha serine/threonine-protein kinase (AKT1), TGF-beta receptor type-2 (TGFBR2), casein kinase II subunit alpha (CSNK2A2), dual specificity protein kinase CLK1 and the aurora kinase C (**Figure 2B**). In line with the results of the bioinformatics pipeline we detected the phosphorylation of the receptor tyrosine-protein kinase erbB-2, which was previously identified as one of the key elements of the hypoxia signature in ovarian cancer cells (23), and of the membrane protein pleckstrin homology-like domain family B member 2 (PHLDB2), which is a known substrate for the protein kinase C (57). In agreement with the regulated pathways (**Figure 2B**, mTOR-AKT1), phosphorylation of AKT2 was consistently repressed. This was accompanied by a robust regulation of rho GTPases pathway (ARHGEF: rho guanine nucleotide exchange factor 12 and dedicator of cytokinesis protein 7, DOCK7) and of the RAF proto-oncogene serine/threonine-protein kinase (RAF1, **Figure 2C**).

Similarly to hypoxia, also application of 3h shear stress stimulation protocol resulted in multiple phosphorylation events in SKOV-3 cells, which could be assigned to cellular components governing cell morphology and adhesion, as well as to the ribonucleoprotein complex (**Figure 2D**). Retracing the hypoxia signature, analysis of the kinase groups primarily involved in these events revealed a coherent picture; albeit different isoforms in comparison to hypoxia, a member of the cyclin-dependent kinase (CDK7) and protein kinase C (PRKCD) were significantly regulated (**Figure 2E**). This is in line with previous studies highlighting the role of CDK7 in regulating ovarian cancer proliferation (58). Specific for the response profile of the physical stimulation protocol was the serine/threonine-protein kinase MARK1, which is deputed to the regulation of cell polarity and involved in the regulation of microtubule growth and turnover (59). Regarding de-phosphorylation events, several analogies could be found with the hypoxia signature (AURKC, CLK1, mTOR, and mitogen-activated protein kinases MAPK-isoforms 9, 13, 3, 8 and 1; **Figure 2E**). Specific of the shear stress signature was the regulation of the RAF proto-oncogene serine/threonine-protein kinase (RAF1), which is known to modulate the expression of tight junctions (60), the MAPK/ERK cascade (61) and the regulation of interferon-induced, double-stranded RNA-activated protein kinase (EIF2AK2, also protein kinase R -PKR). The latter contributes to the tuning of multiple pathways like autophagy (62), interferon response (63) and, in more general terms acts as mediator of cancer cell metabolic plasticity in response to stressors (64). A connection toward the metabolic adaptive potential of SKOV-3 in response to shear stress could be further underpinned by the alteration of pathways associated with the glycogen synthase kinase (GSK) 3b whose inhibition was described to be protective against the onset of myocardial fibrosis and mitochondrial oxidative stress (65). In connection with the application of a physical stimulation protocol, post translational modifications were detected for proteins essential for cell structure and motility (**Figure 2F**). For example, a robust phosphorylation of the motor protein kinesin light chain 2 (KLC2) could be detected. This was accompanied by a dephosphorylation of the anchoring protein AKAP8 which governs the subcellular localization of the PKA and plays a pivotal role in the regulation of the cell cycle (66) and a member of the linker proteins (CLIP1) which regulates dynamics and vesicular transport on the microtubule cytoskeleton (67). In addition, significant dephosphorylation was detected for the oxidation resistance protein 1 (OXR1, involved in the protection from oxidative damage (68), the TP53-binding protein 1, which is involved in the double-strand break repair machinery in response to DNA damage (69) and for ASAP1, a protein which was related to tumor cell adhesion motility and invasiveness *in vitro* and *in vivo* (70).

### Phosphoproteome Profile of OVCAR-3 Cells After 3h Incubation in Reduced Oxygen or Shear Stress

As previously described for SKOV-3 cells, also for OVCAR-3 cells, incubation in reduced oxygen for 3h triggered multiple regulatory events involving cell structural adaptation and affecting the nuclear compartment (**Figure 3A**). Similar to the SKOV-3, the positive phosphorylation events in OVCAR-3 could be associated with the protein kinase C (isoform beta; **Figure 3B**). Intriguingly, three kinase pathways were positively regulated in the OVCAR-3 which were negatively regulated in the SKOV-3 namely MAPK11, CSNK2A1 and mTOR. Positive phosphorylation of the inhibitor of nuclear factor kappa-B kinase subunit beta, which is an essential component of the NF-ĸB signaling pathway, was exclusively detected in OVCAR-3 upon hypoxic conditions. De-phosphorylation events could be associated to the regulatory activity of the serine/threonine-protein kinase NLK, of three different isoforms of the Serine/threonine-protein kinase MARK (1, 3 and 4; **Figure 3B**) and the dual specificity protein kinase TTK, glycogen synthase kinase (GSK) 3b and the serine/threonine-protein kinase A-Raf. Further, changes in phosphorylation profile of several proteins regulating cell shape morphology were observed: among these an increased phosphorylation of caveolae-associated protein 1 (PTRF-CAVIN1, **Figure 3C**) and decreased phosphorylation of the coiled-coil domain-containing protein 6 (CCDC6), src substrate cortactin (CTTN), the scaffold protein scribble homolog SCRIB as well as the catenin delta-1 (CTNND1, **Figure 3C**). In addition, robust de-phosphorylation of the DNA replication licensing factor MCM3 was detected (**Figure 3C**).

As for incubation in reduced oxygen, also shear stress stimulation substantially affected the PTMs in OVCAR-3 cells. Phosphorylation-dephosphorylation events underpinned regulatory functions in the nuclear region, as well as the modulation of structural elements and the interaction with the extracellular matrix (**Figure 3D**). Increased phosphorylation events were attributed to the activation of four main pathways, namely dual specificity mitogen-activated protein kinase kinase 5 (MAPK5), Serine/threonine-protein kinase PAK2, Serine/threonine-protein kinase tousled-like 2 (TLK2) and cGMP-dependent protein kinase 1 (PRKG1). Consistent with the hypoxia signature, the de-phosphorylation events could be traced back to the NLK, MARK1/4 and the casein kinase (I isoform alpha CSNK1A1). Specific for the shear stress stimulation protocol was the involvement of cyclin-dependent kinase 7 (CDK7) and of the serine-protein kinase ATM (**Figure 3E**). The cellular components highlighted by the ORA enrichment analysis (**Figure 3D**, suggestive of regulation of cell structure and proliferation) were coherent with the measured regulatory events: As an example, Rho guanine nucleotide exchange factor 17 (ARHGEF17, **Figure 3F**) and disintegrin and metalloproteinase domain-containing protein 9 (ADAM9, **Figure 3F**) where de-phosphorylated. Indeed, ARHGEF17 was previously reported to be directly involved in mitosis (71) and the expression of ADAM9 was associated with the progression and resistance of several cancer types (72). Very robust increased phosphorylation could be detected for the signal-induced proliferation-associated 1-like protein 3 [SIPA1L3, regulator of cytoskeletal organization and cell polarity (73)], CTP synthase 1 (CTPS1), sequestosome-1 (SQSTM1) and ankycorbin [RAI14, membrane-cytoskeletal linker (74)].

### Multiparametric Morphological Analysis

**Figure 4:**
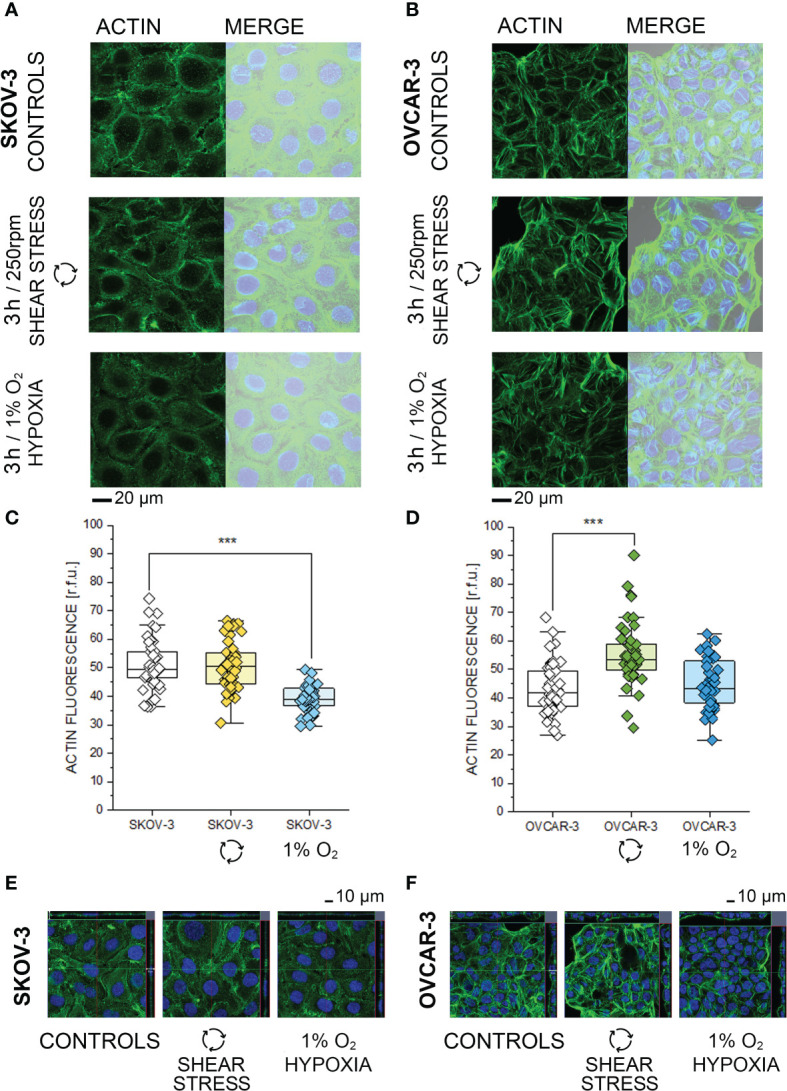
Morphological characterization of SKOV-3 and OVCAR-3 after 3h incubations. **(A, B)** Appearance of actin cytoskeleton (green) and merged image with bright field and cell nuclei (Dapi, depicted in blue). **(C, D)** Quantification of actin cytoskeleton signal *via* image analysis expressed as relative fluorescence units (r.f.u.). Data were obtained from n > 45 cells and significant difference between treatment group is indicated ***p < 0.001 t-Test. **(E, F)** Cross sections from three-dimensional reconstruction of confocal images.

**Figure 5:**
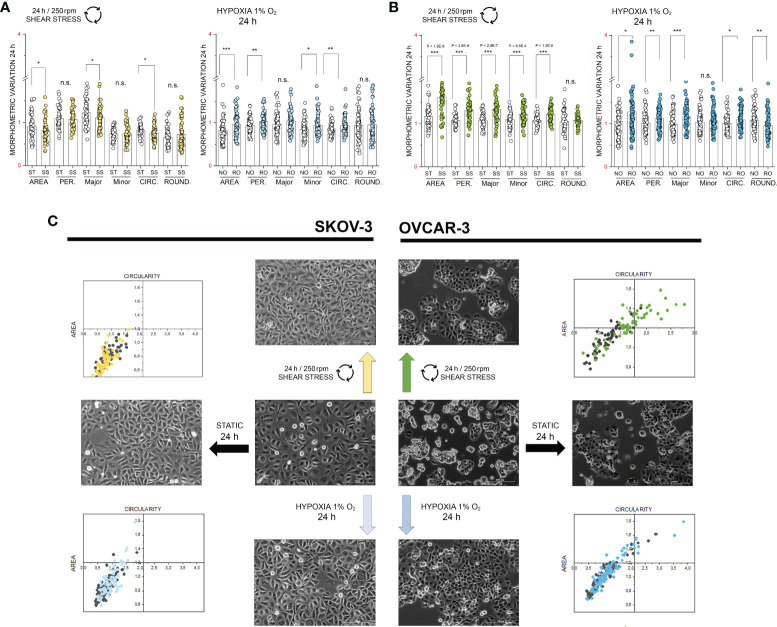
Multiparametric image analysis of the appearance of SKOV-3 and OVCAR-3 cells with or without treatments. **(A)** morphometric variation of SKOV-3 after 24h incubation including area, perimeter, Major/Minor axes, circularity and roundness. **(B)** morphometric variation of OVCAR-3 after 24h incubation including area, perimeter, Major/Minor axes, circularity and roundness. ST static incubation (white diamonds), SS shear stress (250 rpm, 2.8 dyn/cm^2^, light green and yellow diamonds), NO Normal Oxygen (20%, white diamonds); RO Reduced Oxygen (1%, hypoxia, blue and light blue diamonds). Data are normalized to values at the beginning of the experiments (1). n.s., not significant. **(C)** Representative phase contrast images of SKOV-3 and OVCAR-3 cells and mutual behavior of area and circularity morphometric descriptors (grey points controls, colored points treatments). Scale bars stand for 200µm, n = 50 cells, significant difference t-Test *p < 0.05, **p < 0.01, ***p < 0.001.

Based on the phosphoproteome analysis, both SKOV-3 and OVCAR-3 responded to the hypoxia or shear stress stimulation protocols by modulating pathways that are tightly related to morphological adaptation. In order to validate these results with a complementary approach, confocal microscopy experiments were performed. In SKOV-3 fluorescence staining revealed a rearrangement of actin after application of 3h hypoxia protocol. This was visible as a decrease of the density of the cytoskeletal network (**Figure 4A**) and measurable as reduction of actin fluorescence intensity (**Figure 4C**). OVCAR-3 were rapidly responsive to the shear stress stimulation and actin filaments formed stress fibers especially localized at the cell periphery (**Figure 4B**). In agreement, a significant increase of the fluorescent signal could be detected (**Figure 4D**). Three dimensional reconstructions confirmed these readouts and clearly demonstrated that SKOV-3 cells flatten up upon hypoxia incubation and OVCAR-3 re-organize in the vertical dimension in response to shear stress (**Figures 4E, F**). In order to verify the persistence of these responses after longer incubation times, multiparametric image analysis was performed after 24h treatment. Single cells were assessed, considering area, perimeter, minor and major axis, circularity and roundness as reference parameters. Application of 24h shear stress protocol decreased the area, the major axis and the circularity of SKOV-3 cells (**Figure 5A**). Incubation in reduced oxygen conditions increased the size of SKOV-3 cells. This was visible as significant increase of area, perimeter and of the minor axis. This was accompanied by an increased circularity (**Figure 5A**). Cluster analysis of area and circularity revealed a consistent adaptive behavior between controls and treatments (**Figure 5C**). Analysis of the OVCAR-3 revealed a robust increase of almost all shape descriptors (**Figure 5B**). Cluster analysis revealed homogeneous behavior in the circularity/area ratio for the hypoxia incubation in comparison to controls (**Figure 5C**). In accordance to the rapid formation of stress fibers detected after 3h, 24h shear stress stimulation induced the most prominent morphological changes in the OVCAR-3. In this case, it was also possible to observe a sub-clustering of the subpopulations between control cells (grey) and shear stress treated (green; **Figure 5C**).

### Proteome Profile of SKOV-3 and OVCAR-3 After 24h Reduced Oxygen Incubation

**Figure 6:**
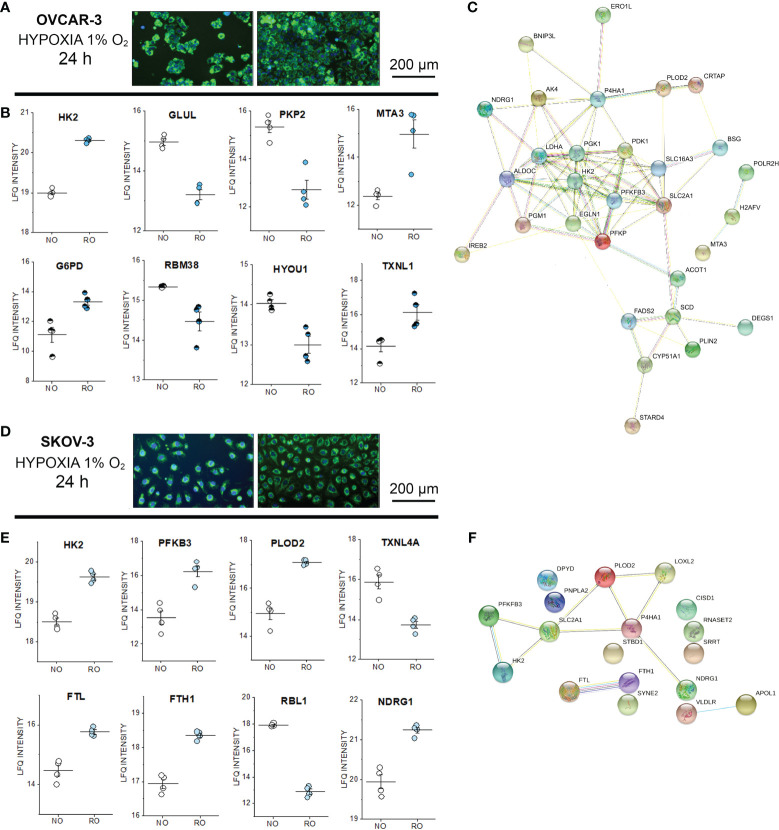
Proteome profile of SKOV-3 and OVCAR-3 cells after 24h hypoxia incubation. Appearance of the cells before and after the experiments **(A)** OVCAR-3 and **(D)** SKOV-3; nuclei counterstained in blue (Hoechst 33258 dil. 1:1000) and mitochondria in green (MitoTracker, dil. 1:1000). **(B)** Representative regulated proteins in OVCAR-3 cytoplasmic fraction (empty circles) and nuclear fraction (half full circles). **(C)** Protein-Protein association clusters obtained with STRING (37) from significantly up-regulated proteins after hypoxia incubation in OVCAR-3. Circles indicate proteins and connection lines are proportional to the confidence of the supporting data describing the interactions. Representative significantly regulated proteins in SKOV-3 cytoplasmic fraction **(E)**. **(F)** Protein-Protein association clusters obtained with STRING (37) from significantly up-regulated proteins after hypoxia incubation in SKOV-3. Circles indicate proteins and connection lines are proportional to the confidence of the supporting data describing the interactions. NO Normal Oxygen (20%, white circles); RO Reduced Oxygen (1%, hypoxia, blue and light blue circles).

In order to verify to what extent incubations in reduced oxygen or shear stress could trigger measurable persistent alterations of the proteome profile, untargeted proteome analysis was performed also after 24h stimulation (**Supplementary Figure S6**). Both cell types endured the hypoxia stimulation protocol without decrease of the cell density (**Figures 6A–D**). Oxygen reduction resulted in a significant alteration of the expression profile of more than 500 proteins in OVCAR-3 cells distributed among nuclear and cytoplasmic fractions (FDR 0.05, **Supplementary Material** and **Supplementary Figure S6**). For SKOV-3 variations were limited to 20 proteins in the cytoplasmic compartment. For the signature of OVCAR-3, deregulation clearly affected proteins dictating metabolic status: this was evident for example in the upregulation of the glycolytic enzyme hexokinase-2 (HK2) and the downregulation of the glutamine synthetase (GLUL). In addition, proteins suggestive of structural remodeling were also modulated such as the junctional protein plakophilin-2 (downregulated) and the metastasis-associated protein MTA3 (upregulated, **Figure 6B**). Consistent with the metabolic adaptive signature of OVCAR-3, glucose-6-phosphate 1-dehydrogenase was upregulated in the nuclear fraction. Tumor suppressor and p53 modulator RBM38 (75, 76) and HYOU1 (Hypoxia up-regulated protein 1) significantly decreased after 24h in reduced oxygen environment (Nuclear fraction, **Figure 6B**). Remarkably, the thioredoxin-like protein 1 was upregulated after hypoxia (**Figure 6B**). In this respect, it was previously demonstrated that upregulation of thioredoxin 1 contributes to drug resistance in ovarian cancer cells (77). In line with the adaptive response of OVCAR-3 to hypoxia, we also observed modulation of several transcription factors, such as TAF1 [regulator of cell cycle (78), downregulated] the aryl hydrocarbon receptor nuclear translocator (ARNT, already reported as tumor growth promoter (79), upregulated) and the steroid hormone receptor ERR1 (ESRRA, downregulated; **Supplementary Figure S7**). As for OVCAR-3, also for SKOV-3 cells we measured a proteome profile shift suggestive of adaption toward glycolysis; consistently an increase of the HK2 in the cytoplasmic compartment (**Figure 6E**) was clearly detectable. In addition, phosphofructokinase (PFKB3) and the procollagen-lysine,2-oxoglutarate 5-dioxygenase 2 (PLOD2) were also upregulated (cytoplasmic fraction, **Figure 6E**). However, in contrast to OVCAR-3, the thioredoxin-like protein 4A (TXNL4A) was reduced/depleted (**Figure 6E**). In addition, ferritin light chain (FTL) and ferritin heavy chain (FTH1) were coherently increased and this reproduces *in vitro* a signature already observed in ovarian tumor biopsies where the increase of ferritin correlates with the progression of the tumor stage (80) and aligns with the emerging role of iron in tumor progression (81). Along this line, other proteins associated with tumor progression were also deregulated: retinoblastoma-like protein 1 (RBL1, downregulated) and NDRG1 protein [necessary for p53 dependent apoptosis, upregulated (82)]. Overall, considering the hypoxia-induced proteome alterations in OVCAR-3 (cytoplasmic fractions), upregulated proteins were found to form a strong molecular network when using the STRING association network analysis software (37) (**Figure 6C**) very much resembling a hypoxia-induced protein cluster recently described for glioblastoma (83). Remarkably, this cluster does exist albeit at much weaker extent in SKOV-3 cells (**Figure 6F**).

### Proteome Profile of SKOV-3 and OVCAR-3 After 24h Shear Stress Incubation

**Figure 7:**
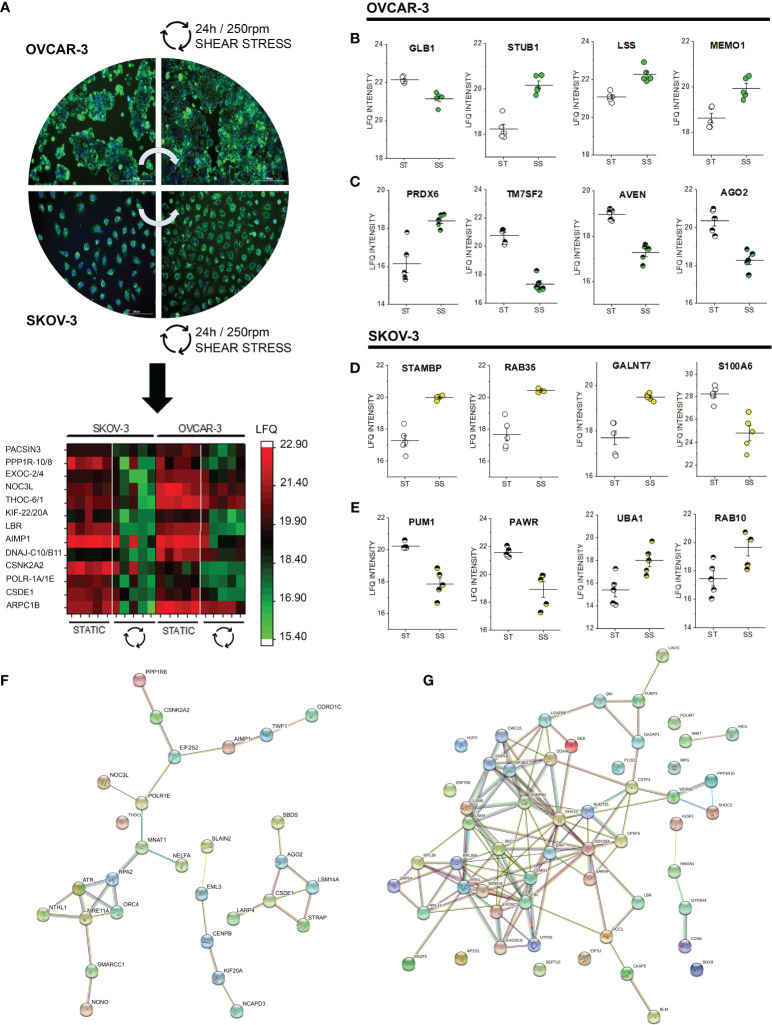
Proteome profile of SKOV-3 and OVCAR-3 cells after 24h shear stress incubation. **(A)** appearance of the cells before and after the experiments; nuclei counterstained in blue (Hoechst 33258 dil. 1:1000) and mitochondria in green (MitoTracker, dil. 1:1000). Heat map summarizes proteins that are significantly regulated (FDR < 0.05) in both OVCAR-3 and SKOV-3. Columns in the heat map are depicting biological replicates (n = 5). Representative significantly regulated proteins in OVCAR-3 cytoplasmic fraction **(B)** and nuclear fraction **(C)**. Representative significantly regulated proteins in SKOV-3 cytoplasmic fraction **(D)** and nuclear fraction **(E)**. ST static incubation (white circles), SS shear stress (250 rpm, 2.8 dyn/cm^2^, yellow and green circles). Protein-Protein association clusters obtained with STRING (37) from significantly down-regulated proteins after shear stress incubation **(F)** OVCAR-3 and **(G)** SKOV-3 cells (nuclear fractions). Circles indicate proteins and connection lines are proportional to the confidence of the supporting data describing the interactions.

After 24h shear stress stimulation, the commonalities detected in the phospho-proteome signature of SKOV-3 and OVCAR-3 (affecting in both cases structural elements and the ribonuclear complex, **Figures 2**, **3**) consolidated in changes of the abundance level of several proteins. Among these, we detected in the nuclear extracts coherent significant regulation of proteins governing the translation machinery (CSDE1, POLR1A, DnaJ homolog subfamilies C10/B11; AIMP1; THOC6; NOC3L) or supporting posttranslational modifications (EXOC2; Serine/threonine-protein phosphatase 1 regulatory subunit 10-PPP1R10, protein kinase C and casein kinase substrate in neurons protein- PACSIN3 and casein kinase II subunit alpha, **Figure 7A** nuclear extracts). Protein association networks analysis distinguished three clusters in OVCAR-3 cells related to cell cycle checkpoint, microtubule-binding proteins and poly(A)-binding proteins (**Figure 7F**), with only the latter partially reproduced in SKOV-3 cells (**Figure 7G**). In addition, for both OVCAR-3 and SKOV-3, consistent regulations could be observed for delta(14)-sterol reductase [LBR, regulator of cholesterol biosynthesis (84)], kinesin-like proteins (KIF-22/20A) and actin-related protein 2/3 complex subunit 1B (**Figure 7A** nuclear extracts). In addition to common regulatory events, SKOV-3 and OVCAR-3 displayed also an individual signature; in OVCAR-3 physical stress lead to a significant downregulation of the beta-galactosidase (GLB1, **Figure 7B**) and upregulation of STUB1 [involved in protein ubiquitination and quality control, (85)], LSS (lanosterol synthase) and MEMO1, the latter was already describe to play an important role in cancer cell motility (86). In parallel, peroxiredoxin-6 (PRDX6) were robustly upregulated in the nuclear fraction. Coherently with the effect on the lipid biosynthesis machinery (LBR, **Figure 7A**), also the delta(14)-sterol reductase TM7SF2 was substantially downregulated. In addition, cell death regulator AVEN and protein argonaute-2 (AGO2, **Figure 7C**) decreased. Also for SKOV-3 cells, application of shear stress resulted in the regulation of proteins governing motility and metabolism. Concerning the cytoplasmic fraction, only 4 proteins were significantly regulated: STAM-binding protein which is involved in the regulation of ubiquitin-dependent receptors trafficking (87) and the Ras-related protein Rab-35 (RAB35), which is involved in endocytic recycling at membrane level necessary for cytokinesis (88), the N-acetylgalactosaminyltransferase 7 (GALNT7) and the S100A6 protein whose expression is deregulated in several cancer types and governs cell proliferation as well as motility (89). In the nuclear fraction, shear stress stimulation protocol additionally decreased the presence of pumilio homolog 1 (PUM1), which is involved in cell replication and genomic stability (90). In addition, a downregulation of PARW (PRKC apoptosis WT1 regulator), whose reduction in tissues was recently described to have a prognostic value for ovarian cancer progression (91) was detected. On the other hand, the ubiquitin-activating enzyme UBA1 [essential for the DNA damage response pathway (92)] was the most prominently upregulated protein in this dataset. In agreement with the upregulation of RAB35 in the cytoplasmic fraction, another member of the small GTPases Rab (RAB10) was found upregulated in the nuclear extracts (93).

## Discussion

In the peritoneal cavity, ovarian cancer cells experience a complex physical and chemical environment. Based on this, it is possible to hypothesize that a constant adaptive pressure could support genetic factors and foster the aggressive phenotype of ovarian cancers. It was previously demonstrated that fluid shear stress can support genomic instability (17), and may thus favor malignant progression. Recent literature identifies in the response to mechanical stimuli, such as those deriving from substrate topology (94) or shear stress (19, 20, 95), a potential for adaptive booster, fostering epithelial-mesenchymal transition and chemoresistance development. However, shear stress can also synergistically support drug efficacy *in vitro* (96). Hence, available data are far from being conclusive and jeopardize the possibility of a clear line of interpretation. In this study we systematically explored the response of SKOV-3 and OVCAR-3 cells to hypoxia and shear stress. Reduced oxygen and biomechanical stimulation are representative of chemical and physical cues in the tumor microenvironment and, being integral physiological components of the peritoneal cavity, have the possibility to imprint on cell phenotype even before therapy. Cultivated ovarian cancer cells SKOV-3 and OVCAR-3 differ clearly in structure and proteome (**Figure 1**). This reflects *in vitro* some crucial features of their pathological behavior *in vivo*: SKOV-3 are classified as non-serous ovarian cancer cells and are characterized by high motility (24). In line, in SKOV-3 cells cytoskeletal elements like tubulins, vimentin and dynamins are expressed at much higher levels (**Figure 1**). On the other hand, OVCAR-3 cells model high grade serous ovarian cancer growing in the peritoneal cavity, a condition which requires shear stress endurance.

### Hypoxia Signature

For the hypoxia signature, we observed for both cells types a metabolic switch toward glycolysis accompanied with characteristic morphological changes. Along this line, it was previously described that hypoxia can trigger vasculogenic mimicry (VM) in ovarian cancer cells (97). VM supports biochemical and morphometric adaptation in non-endothelial cells resembling those of the vascular network and involves RhoA/ROCK, Rac1/PAK pathways as well as EMT (Epithelial Mesenchymal Transition, characterized by e.g. vimentin expression) (98). This would explain why hypoxia incubation triggered distinctive structural reorganization (**Figures 4** and **5**) and mobilized largely cytoskeletal elements in our experimental conditions. In line, in SKOV-3 we observed an overexpression of vimentin in comparison to OVCAR-3 (**Figure 1** and **Supplementary Figure S2**) and, upon treatment, a significant dephosphorylation of Rho guanine nucleotide exchange factor 12 (ARHGEF12, **Figure 2C**), which was previously described to support hypoxia-induced cell migration and proliferation in pulmonary artery smooth muscle cells (99). Consistently, Rho GTPases exchange factor protein Dock7 was also regulated (**Figure 2C**). Dock proteins tune cytoskeletal dynamics and cell morphology (100) as well as Raf-1 which associates to Rho in the regulation of cell migration (101). Intriguingly, oxygen reduction in OVCAR-3 decreased the phosphorylation of delta catenin (CTNND1, **Figure 3C**), which was previously reported to promote lymphangiogenesis and metastatic progression *via* Rho-GTPase dependent mechanism (102). Moreover, as suggested also by our data (**Figure 3C**) delta catenin binds to cortactin (103) and can regulate RhoA GTPase *via* a glutamate dependent mechanism as described in neuronal cells (104). In line, after 24h in reduced oxygen, OVCAR-3 displayed a significant reduction of Rho guanine nucleotide exchange factor 11 (ARHGEF11) and the Rho GTPase-activating protein 10 (ARHGAP10, **Supplementary Figure S7**). Hence, albeit with a different strategy in comparison to SKOV-3, also OVCAR-3 seem to be able to respond to hypoxia with the modulation of pathways downstream inward from the cell membrane to the cytoskeleton. This interpretation could also explain some analogies in the structural remodeling after 24h (**Figure 5**). Albeit on two cell lines and in controlled *in vitro* conditions, the adaptive strategies underpinned by the phosphoproteome profile, consolidated at longer incubation times in significant regulatory events measurable at proteome level. For example, for OVCAR-3 positive phosphorylation events could be associated to increased activity of the PKC (**Figure 3B**). PKC regulates, among others, the transcription of CYP1A1 genes *via* aryl hydrocarbon receptor (AhR) (105). In agreement, we measured a significant increase of the aryl hydrocarbon receptor nuclear translocator (ARNT, nuclear fraction hypoxia treatment, **Supplementary Figure S7**), implying that reduced oxygen can modify metabolic competence of OVCAR-3. ARNT is essential for the coordination of the hypoxia inducible factor (HIF) and respective metabolic transition toward glycolysis (106). ARNT was recently found upregulated in clear cell renal cell carcinoma: its expression was responsible for cell migration/invasion and, coherently with our data (**Figure 6**) also for the regulation of glycolytic enzymes like PFKFB3, (6−phosphofructo−2−kinase/fructose−2,6−bisphosphatase 3) and HK2 (hexokinase-2) (79). Phosphorylation profiles associated to MAPK11, CSNK2A1/2 and mTOR were regulated in different directions in the two cell types (positively OVCAR-3 and negatively SKOV-3, **Figures 2B** and **3B**). It is worth noticing that this reflects the basal differences of the two models where, even without stimulations, autophagy (**Figure 1B**) and casein kinases isoforms (**Figure 1F**) were divergently balanced. Along this line, it was previously described that resistant ovarian cancer cells have high metabolic plasticity and are even able to switch “on demand” between glycolysis and OXPHOS (107). In this respect, casein kinase 2 can drive metabolism of cancer cells *via* manifold interactions including *i)* Wnt/β-catenin, *ii)* PI3K/Akt/mTORC1, and *iii)* p53/HIF-1α (108); all these elements were significantly regulated in our models, possibly supporting the interpretation that hypoxia could foster the abovementioned metabolic levers. As an example, β-catenin was described to tune glutamine metabolism (109) and in OVCAR-3 we observed significant deregulation of the targets of the kinase NLK [Wnt/ß-Catenin pathway (110)] after 3h and a downregulation of the glutamine synthetase (GLUL) after 24h (**Figure 6B**). Of note, even if regulatory events triggered by hypoxia were more limited in SKOV-3 when compared to OVCAR-3 (**Figure 6**), they maintained high biological relevance with respect to pathophysiological progression of the disease. Along this line, we observed for instance pronounced upregulation of ferritin light and heavy chains (FTL and FTH1, **Figure 6E**): it was recently demonstrated that expression levels of FTL and FTH1 significantly correlate with immune cells infiltration and poor overall patients survival in several cancer types, including the ovarian serous cystadenocarcinoma (111).

### Shear Stress Signature

In line with the putative pathophysiological behavior of SKOV-3 and OVCAR-3, the high-grade serous cell model OVCAR-3 adapted to shear stress protocol with prompt structural remodeling. Morphometric descriptors such as area and circularity suggested OVCAR-3 to be particularly responsive to physical cues (**Figure 5**) and formation of actin stress fibers could be observed already after 3h incubation (**Figure 4B**). This is in line with previous data describing force-dependent cytoskeletal reorganization potential for these cells (16) and typical growth of this tumor type in the peritoneum. In comparison to SKOV-3, cholesterol biosynthesis pathway components were constitutively upregulated in OVCAR-3 (**Figure 1F**) together with mitochondrial proteins (**Figures 1B, C**) and calcium management proteins (**Figure 1D**). It was previously described for endothelial cells that shear stress can induce mitochondrial OXPHOS by depleting cell membrane cholesterol (112) and physical stress can sustain calcium signaling and ATP production (113, 114). From this perspective, the adaptive potential of OVCAR-3 seems to retrace physiological strategies already used by other cell types (e.g. endothelial cells). Along this line, application of shear stress (LSS; 5 dyn/cm^2^) downregulates lipid metabolism in human umbilical vein endothelial cells (115). In our model, upon application of shear stress (24h) we could confirm a robust regulation of proteins essentials for cholesterol biosynthesis (delta(14)-sterol reductase TM7SF2 and LBR; lanosterol synthase LSS, **Figures 7A–C**), and this response was more pronounced in OVCAR-3. In addition, the cytoskeletal liker protein RAI14, which was consistently phosphorylated (3h, **Figure 3F**) and upregulated in OVCAR-3 after shear stress, was previously described in relation to its capacity to foster the proliferation of breast cancer cells (116) and the progression of gastric cancer (117). In our experimental conditions also SKOV-3 responded to physical cues adapting the proteome signature, and, consistently, displaying some indications of a more aggressive phenotype. This includes, among others, the upregulation of RAB10 (**Figure 7E**), which was recently suggested as novel therapeutic target for ovarian cancer in virtue of its activity as regulator of cell migration (118) or the downregulation of PAWR whose loss is considered a clinical biomarker for ovarian cancer cell progression (91). However, the shear stress stimulation protocol also decreased expression of the CSNK2A2 (OVCAR-3 and SKOV-3; **Figure 7A**): the same target was identified among a gene expression panel correlating with ovarian cancer progression *in vivo* and *in vitro*, hence supporting the complexity of the physically induced signature and the difficulty to draw conclusions on the bases of one single protein or target. A recent study comparing SKOV-3 and OVCAR-8 in response to constant or oscillatory strain revealed that the two cell types adapt differentially to the two tensional protocols (119). Particularly, oscillatory tension (OT) increased migration in both cell types and constant tension (CT) only in the OVCAR-8. Similarly, mechanical strain increased proliferation, but exclusively in SKOV-3 cells. Overall this suggests that ovarian cancer cells are sensitive to mechanical cues, and can discriminate among stimulation types, as previously described also for endothelial cells [laminar vs. oscillatory shear stress (120)]. In general, this also agrees with previous studies describing heterogeneity of cancer cell models supposedly depicting the same pathological condition (27). Keeping these considerations in mind, we observed a limited number of proteins which was coherently regulated in both cell types (**Figure 7**). However, cellular components affected by shear stress were relatively consistent in both phosphoproteome datasets (**Figures 2D**, **3D**), and, to some extent, in the protein networks associations (**Figures 7F, G**). Intriguingly, more than 40 proteins which were regulated in SKOV-3 upon shear stress were regulated in OVCAR-3 after hypoxia (**Supplementary Figure S8**). This includes proteins regulating cell proliferation and could complement the observations of Martinez and colleagues on the differential proliferation of SKOV-3/OVCAR-8 after application of substrate strain (119). Indicative was, among others, the regulation of the TEAD1/2 transcription factors, which governs *via* the Hippo pathway cellular proliferation and EMT (121). Following the same line of interpretation, also in the phosphoproteome data we could observe that protein kinase C substrates were phosphorylated in OVCAR-3 upon hypoxia (**Figure 3B**, beta isoform) and in SKOV-3 upon shear stress (**Figure 2E**, delta isoform).

## Conclusions

Overall, adaptive strategies to hypoxia and shear stress of SKOV-3 only partially overlap with those of OVCAR-3 or are levered with a different kinetic. These strategies could also mirror complementary molecular mechanisms governing inside-out (hypoxia driven) or outside-in (shear stress driven) cell response, as for instance occurring in the tumor core or on its surface. At cellular level, considering the changes in the metabolic competence, including lipid metabolism, and cytoskeletal remodeling targeting the cell surface, the plasma membrane appears to serve as an integration site for stimuli of chemical and physical origin. In conclusion, we identified crucial elements guiding structural and metabolic adaptation of OVCAR-3 and SKOV-3 upon shear stress and hypoxia. We described mechanical cues as contributing factors in driving adaptive potential of the two cell types and created a model that paves the way for further investigation on the role of chemical-physical signals in the progression of ovarian cancer.

## Data Availability Statement

Mass spectrometry phosphoproteomics data have been deposited to the ProteomeXchange Consortium *via* the PRIDE (122) partner repository with the dataset identifier PXD027466. Complete list of regulated proteins after 24h incubation (hypoxia or shear stress incubation) is provided as **Supplementary Material**. Peptide sequence and phosphorylation sites used to generate **Figures 2** and **3** are provided as **Supplementary Material**.

## Author Contributions

AB, PB, MK, LJ, and EK performed experiments, data analysis and statistics. CG and GDF conceptualized the study and provided resources. GDF wrote the original draft of the manuscript. All authors reviewed, edited and approved the manuscript.

## Funding

This work, including open access publication, was supported by the University of Vienna (intramural funding).

## Conflict of Interest

The authors declare that the research was conducted in the absence of any commercial or financial relationships that could be construed as a potential conflict of interest.

## Publisher’s Note

All claims expressed in this article are solely those of the authors and do not necessarily represent those of their affiliated organizations, or those of the publisher, the editors and the reviewers. Any product that may be evaluated in this article, or claim that may be made by its manufacturer, is not guaranteed or endorsed by the publisher.
