# Supplementary material for: Inward Outward Signaling in Ovarian Cancer: Morpho-Phospho-Proteomic Profiling Upon Application of Hypoxia and Shear Stress Characterizes the Adaptive Plasticity of OVCAR-3 and SKOV-3 Cells
Source: Front Oncol. 2022 Feb 14;11:746411. doi: 10.3389/fonc.2021.746411 (PMC8896345; doi:10.3389/fonc.2021.746411)

Inward outward signaling in ovarian cancer: morpho-phospho-proteomic profiling upon application of hypoxia and shear stress characterizes the adaptive plasticity of OVCAR-3 and SKOV-3 cells.

Andrea Bileck[1,2], Patricia Bortel[1], Michelle Kriz[1,3], Lukas Janker[1], Endre Kiss[4], Christopher Gerner[1,2,4]†, Giorgia Del Favero[3,4]†

1 Department of Analytical Chemistry, Faculty of Chemistry University of Vienna, Vienna, Austria

2 Joint Metabolome Facility, University of Vienna and Medical University of Vienna, Vienna, Austria

3 Department of Food Chemistry and Toxicology, Faculty of Chemistry University of Vienna, Vienna, Austria

4 Core Facility Multimodal Imaging, Faculty of Chemistry University of Vienna, Vienna, Austria

†These authors have contributed equally to this work and share senior authorship and correspondence

(G. Del Favero giorgia.del.favero@univie.ac.at and C. Gerner christopher.gerner@univie.ac.at)

Keywords: Ovarian cancer, fluid shear stress, hypoxia, morpho-metabolic plasticity, vasculogenic mimicry

Figure S1.

Comparison of the proteome profile of OVCAR-3 and SKOV-3 cytoplasmic and nuclear fraction. Overexpression enrichment analysis (ORA) was performed with WebGestalt platform (Liao et al., 2019) biological processes (blue bars) and cellular components (grey bars) of the 250 most regulated proteins between the two cell types.

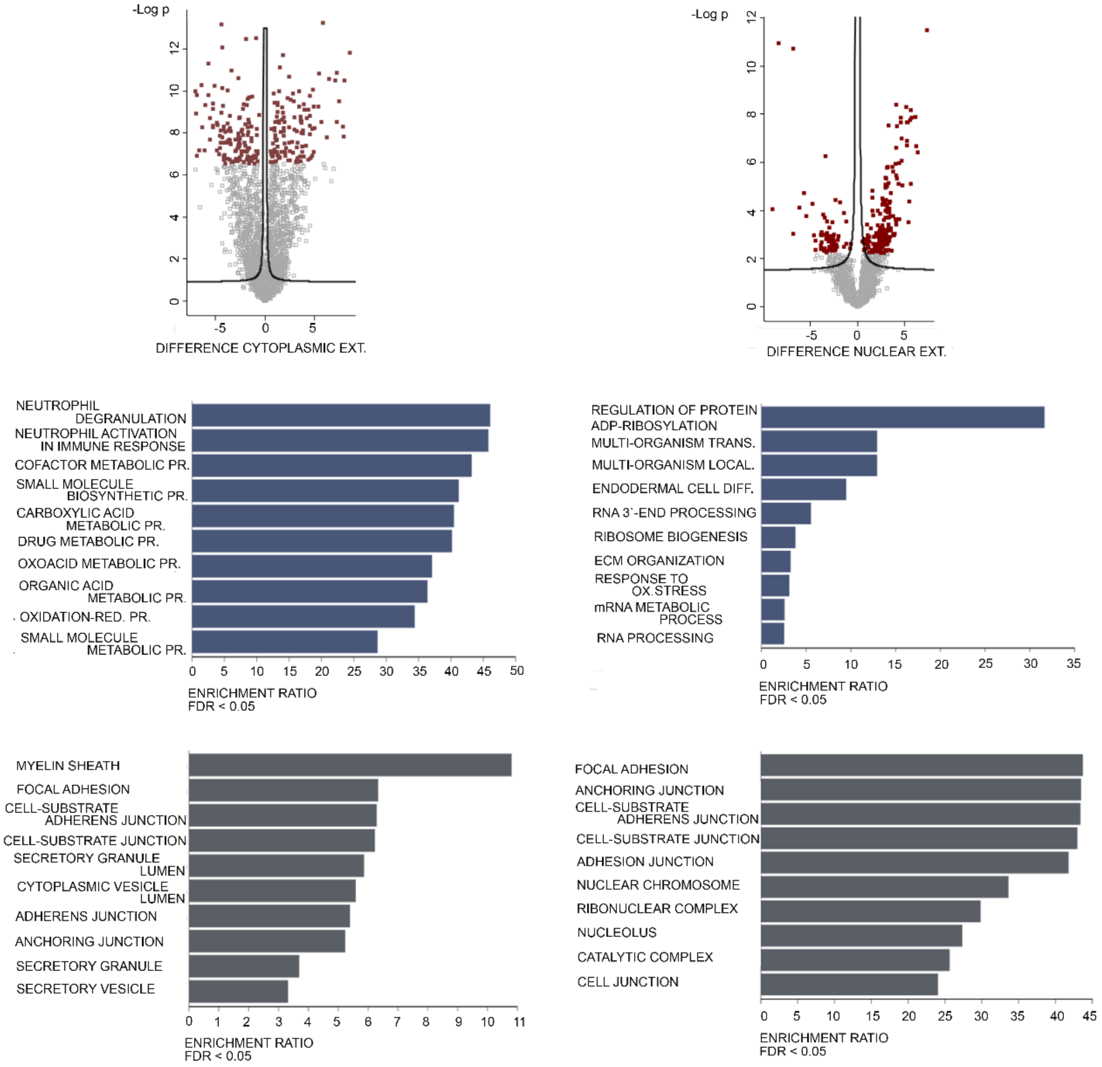

Figure S2.

Comparison of the proteome profile of OVCAR-3 and SKOV-3 cytoplasmic fraction selected cytoskeletal elements (heat map) and selected marker proteins. Color coding are indicative of the LFQ intensities detected during the measurements. Graphs are summarizing epithelial marker proteins such as KRT18, Claudins 3 and 6, EPCAM upregulated in OVCAR-3 (rose symbols) and levels of stem cell marker proteins CD44, L1CAM and KRT19 as well as EMT marker vimentin, ERBB2 and IGFBP7 upregulated in SKOV-3 (green symbols).

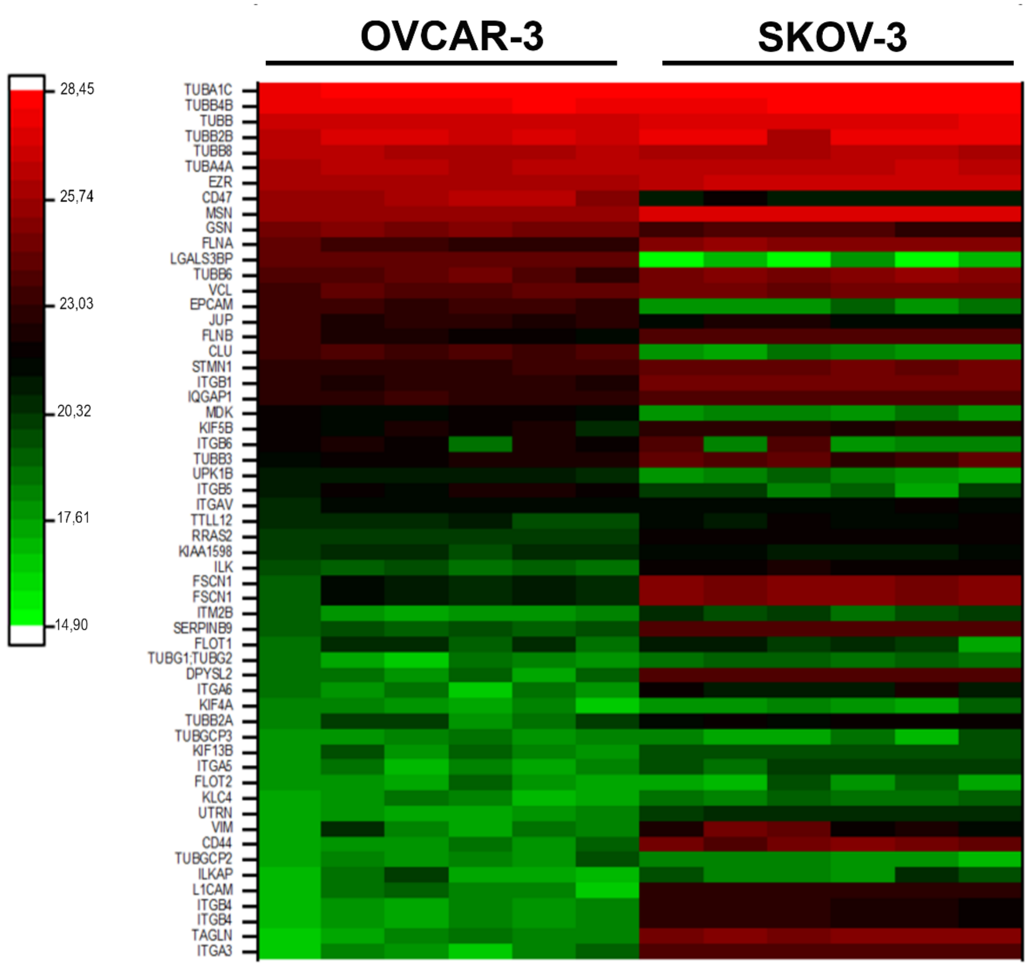

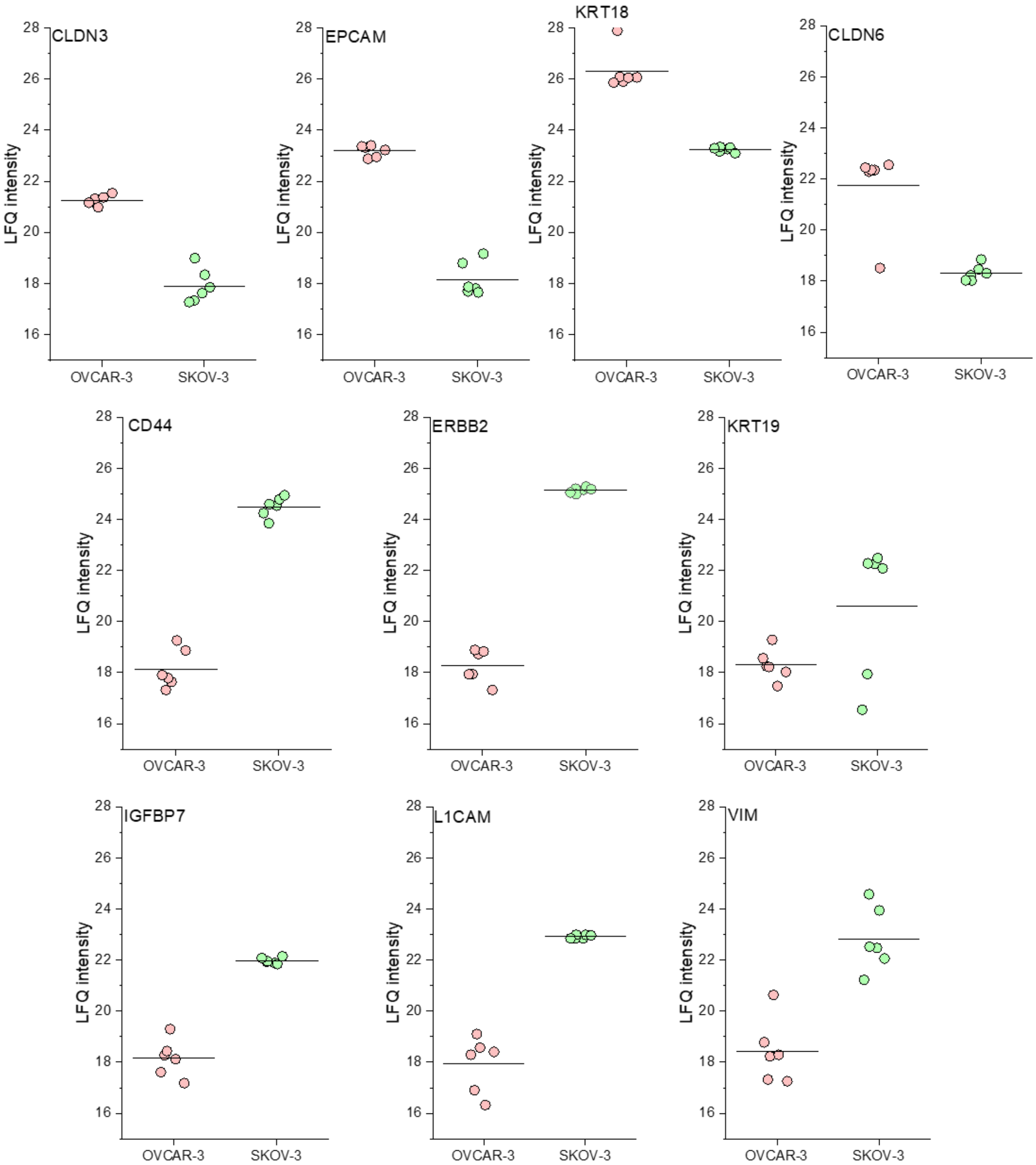
CLDN3
EPCAM
KRT18
CLDN6
CD44
ERBB2
KRT19
IGFBP7
L1CAM
VIM
LFQ intensity
28
26
24
22
20
18
16
OVCAR-3
SKOV-3

Figure S3. Basal Proteome SKOV-3 after 3h incubation in reduced oxygen (hypoxia 1% O2) or shear stress 250 rpm as measured in parallel to the phosphoproteome analysis (Figure 2)

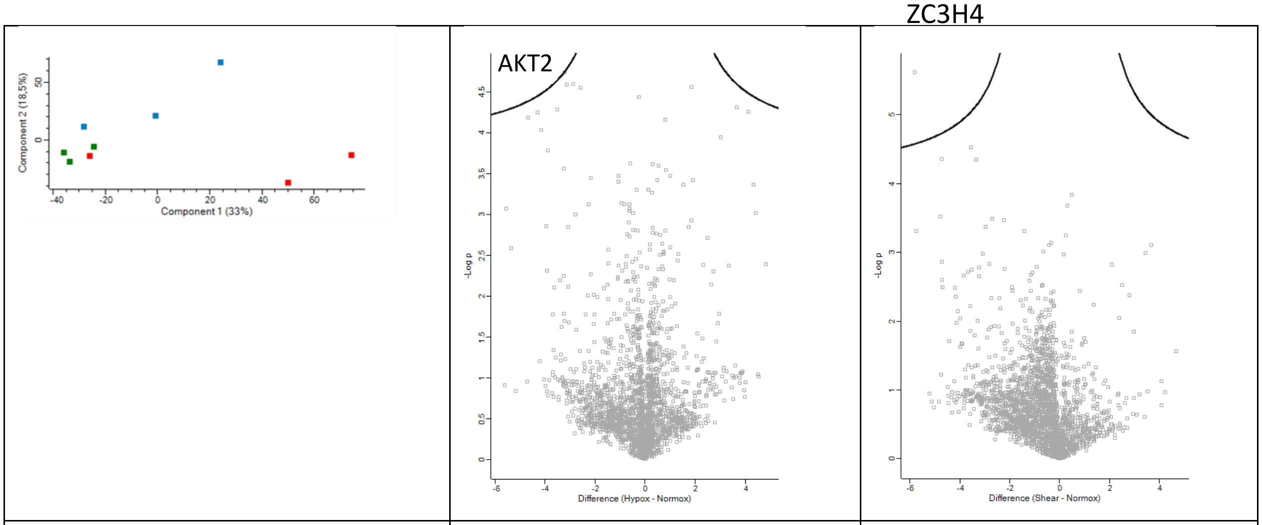

**PCA Normoxia -static Hypoxia 1%O2 Shear stress 250 rpm**	**Difference Hypoxia 1%O2 vs Normoxia -static**	**Difference Shear stress 250 rpm vs Normoxia -static**

Figure S4. Basal Proteome OVCAR-3 after 3h incubation in reduced oxygen (hypoxia 1% O2) or shear stress 250 rpm as measured in parallel to the phosphoproteome analysis (Figure 3).

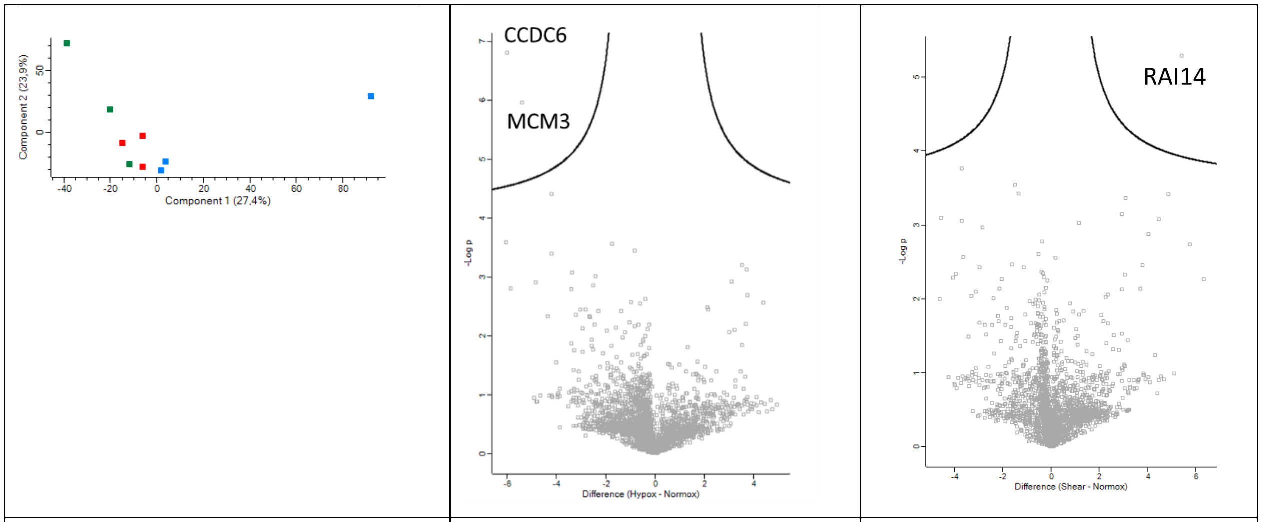

**PCA Normoxia -static Hypoxia 1%O2 Shear stress 250 rpm**	**Difference Hypoxia 1%O2 vs Normoxia -static**	**Difference Shear stress 250 rpm vs Normoxia -static**

Figure S5. Kinase enrichment analysis

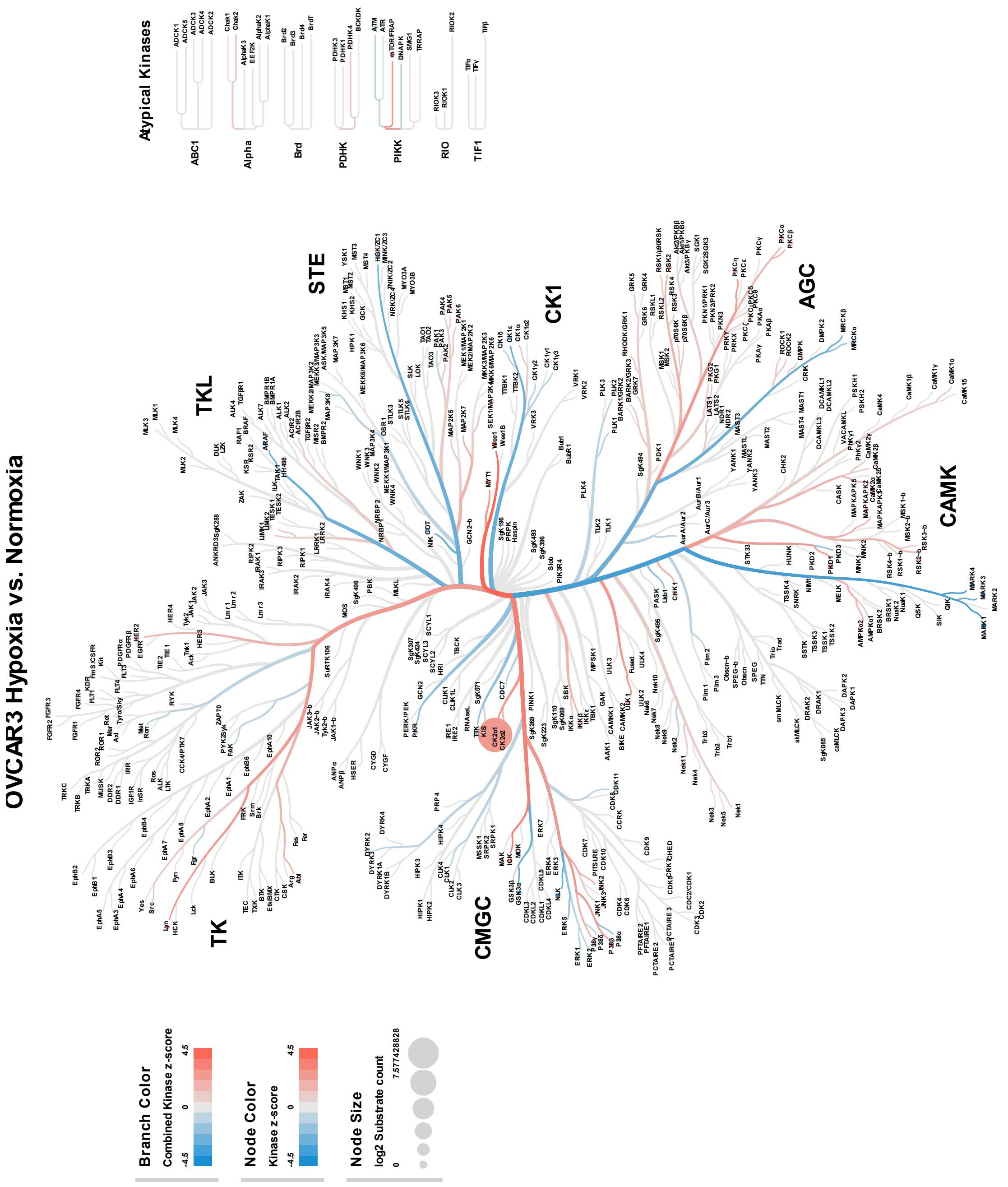

OVCAR shear vs. normox

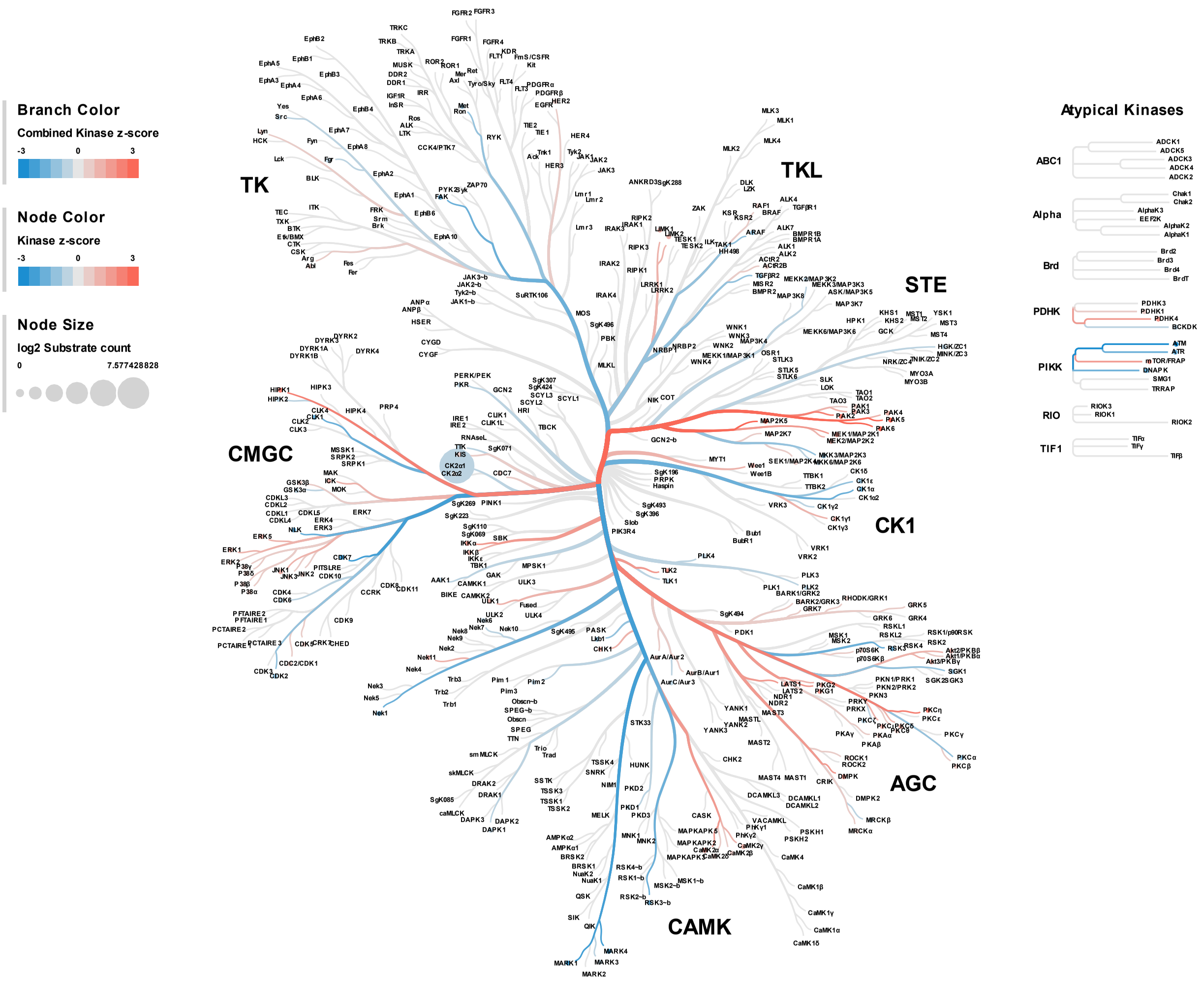

SKOV3 Hypoxia vs. Normoxia

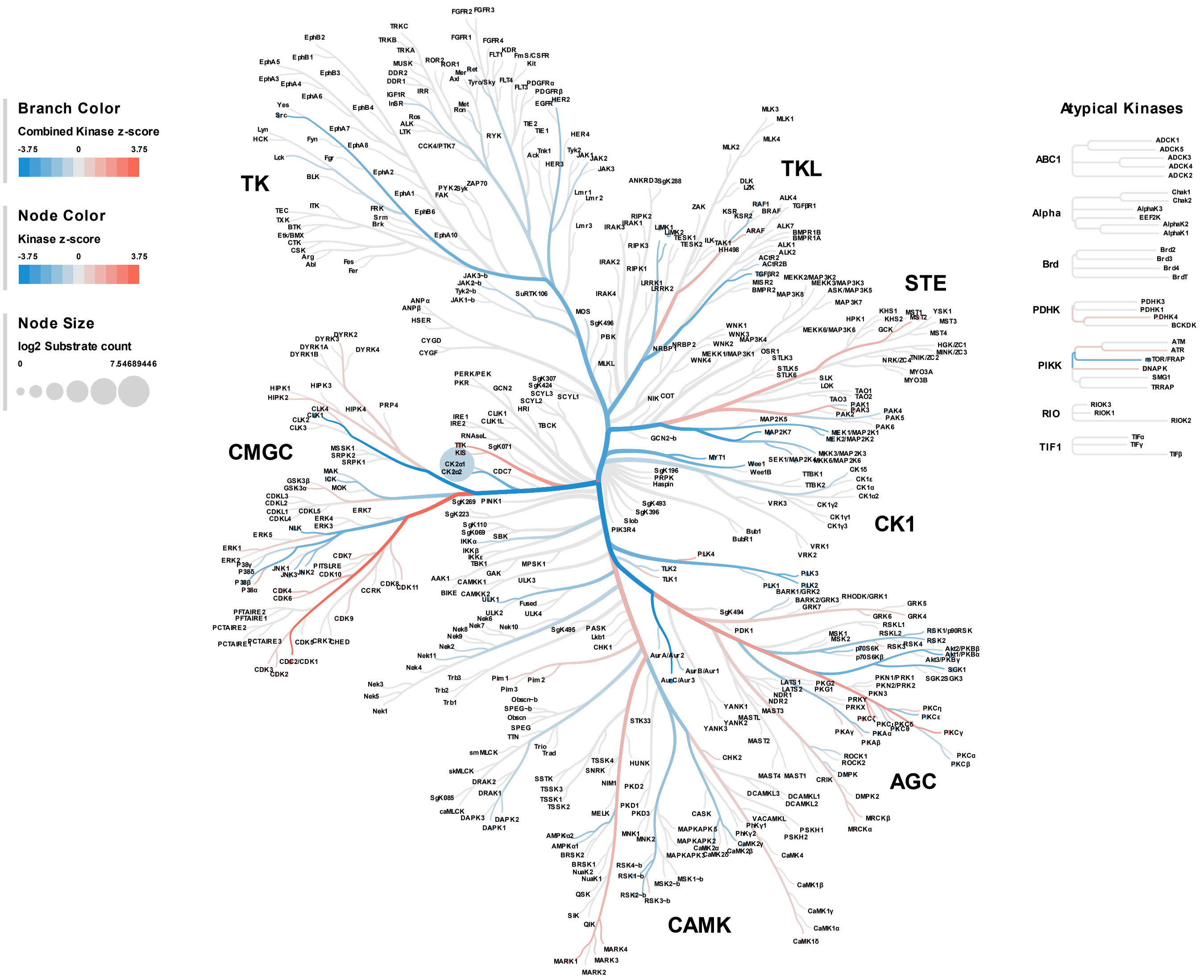

SKOV3 Shear vs. Normoxia

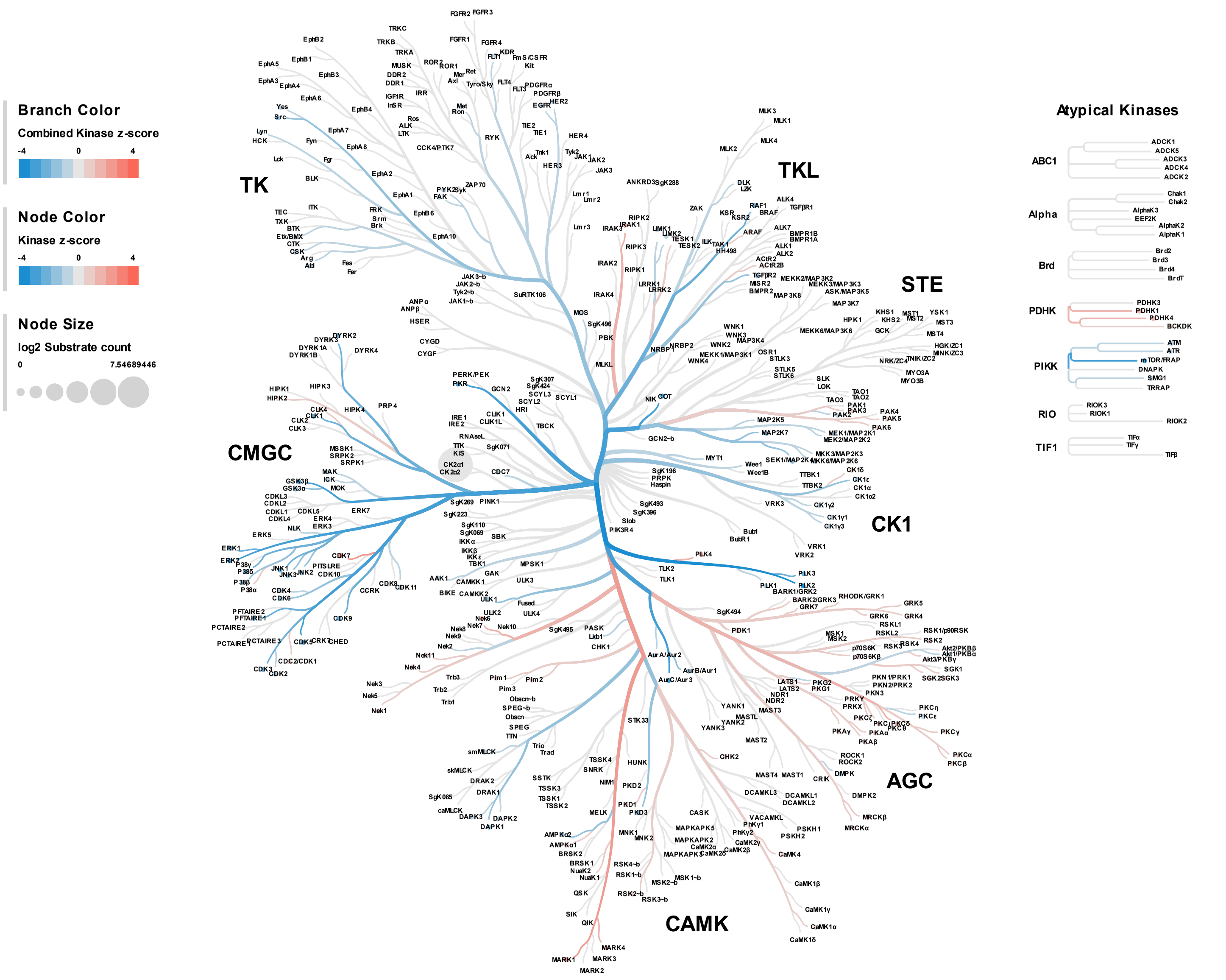

Figure S6. Volcanos PLOT and PCA summarizing proteome after 24h stimulation (FDR 0.05).

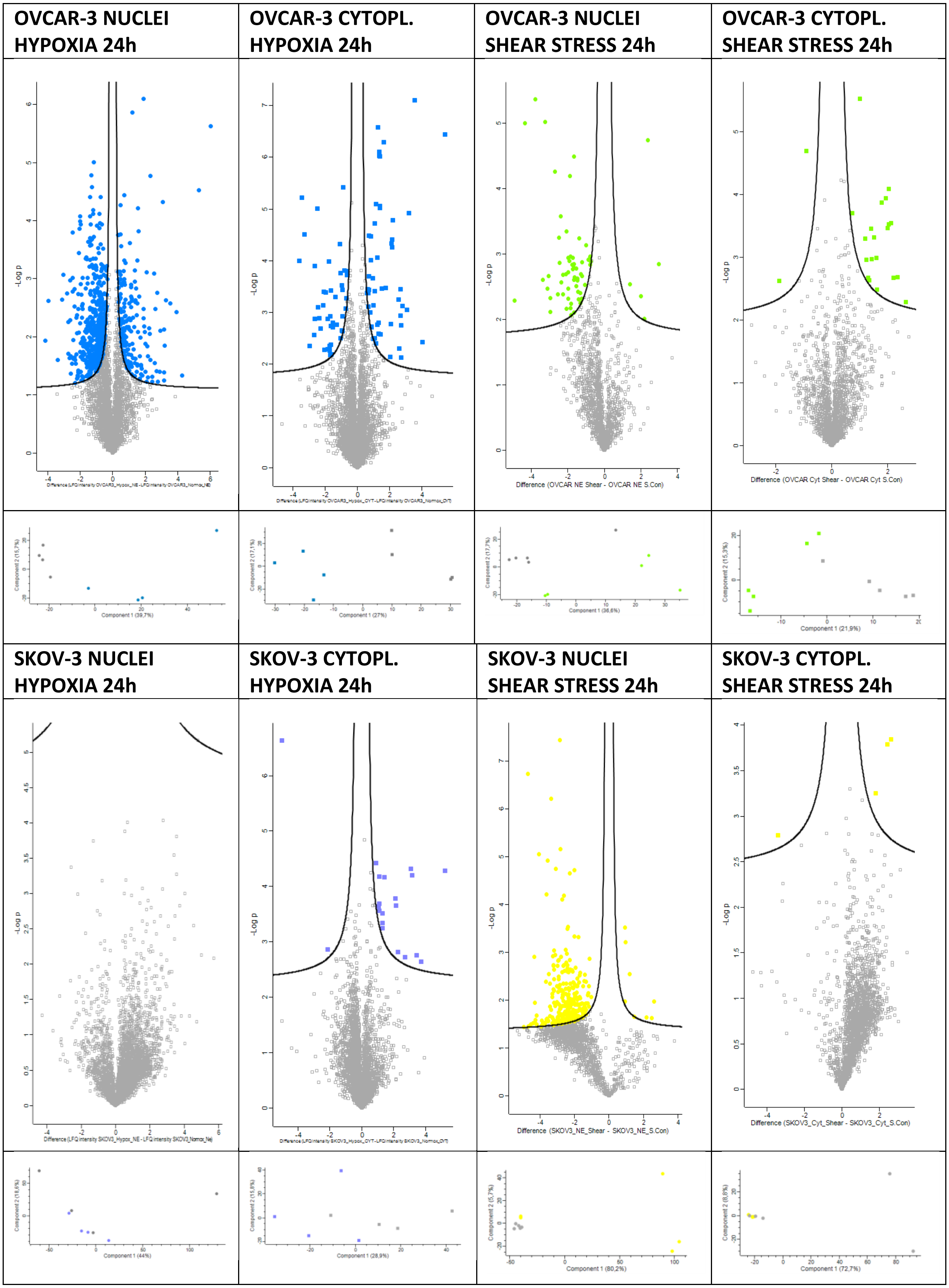

Figure S7.

Additional selected proteins significantly regulated in OVCAR-3 nuclear extracts after 24h incubation in reduced oxygen (hypoxia). NO Normal Oxygen (20%, white circles); RO Reduced Oxygen (1%, hypoxia, blue and light blue circles).

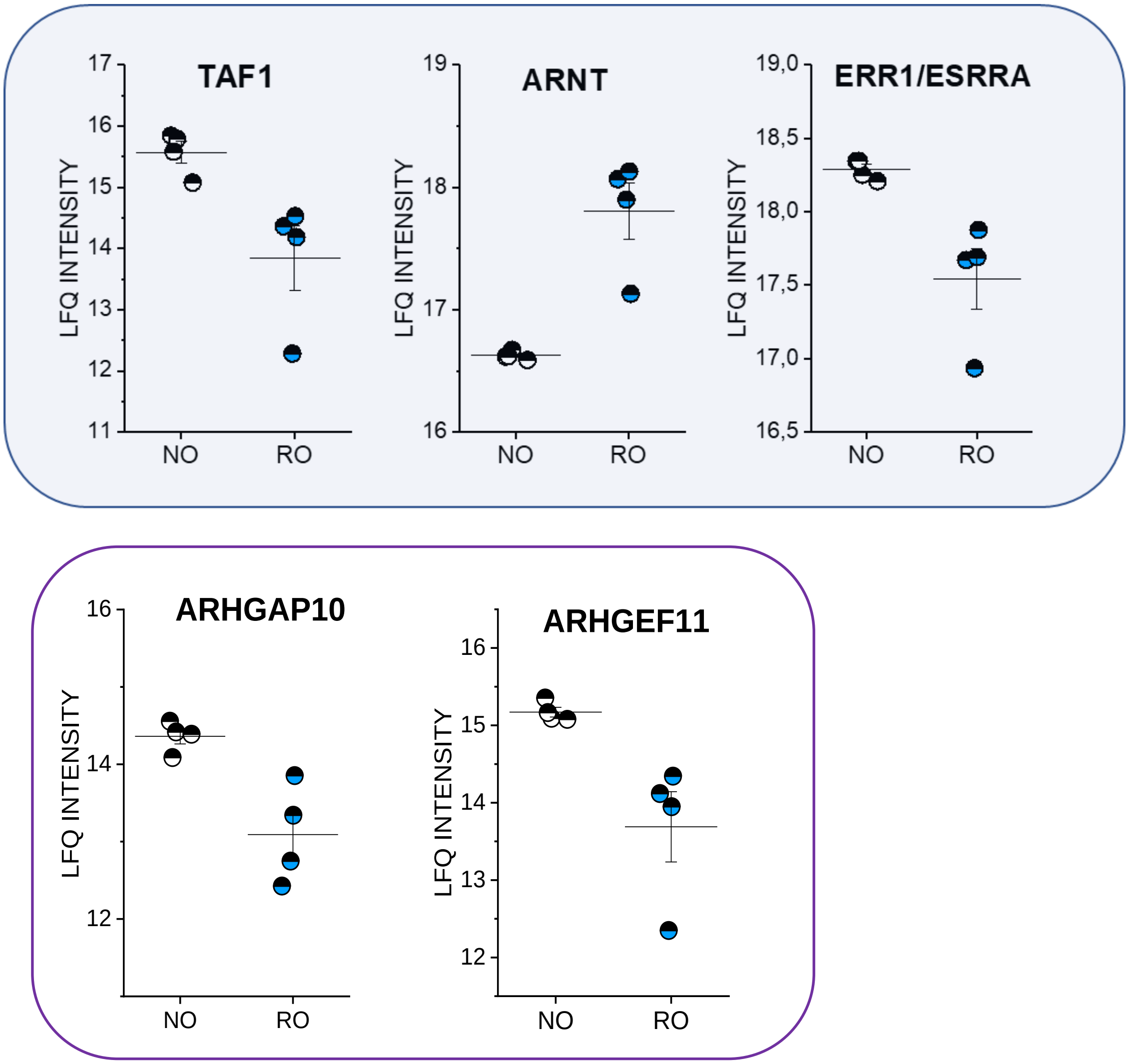

Figure S8.

Proteins significantly regulated in both SKOV-3 and OVCAR-3. Orange squares indicates significant regulation in comparison to respective controls (static, normal oxygen), white squares indicate no regulation. In case multiple isoforms of the same family of proteins was regulated this is indicated with -multiple.

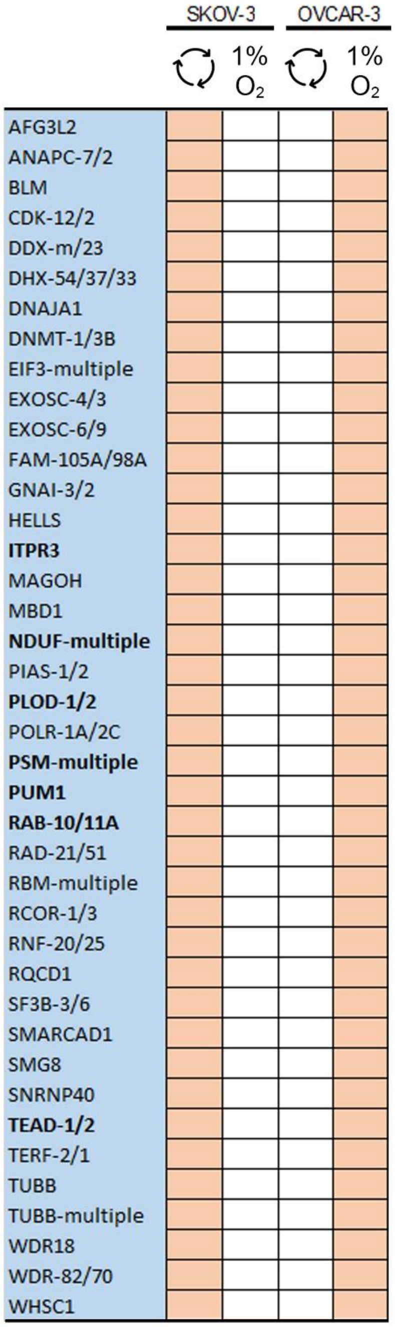

Supplement: Supplementary file 1 [file DataSheet_1.pdf]
